# A comparative study of flow cytometry‐sorted communities and shotgun viral metagenomics in a Singapore municipal wastewater treatment plant

**DOI:** 10.1002/imt2.39

**Published:** 2022-07-28

**Authors:** Xiaoqiong Gu, Yi Yang, Feijian Mao, Wei Lin Lee, Federica Armas, Fang You, David M. Needham, Charmaine Ng, Hongjie Chen, Franciscus Chandra, Karina Yew‐Hoong Gin

**Affiliations:** ^1^ Department of Civil and Environmental Engineering National University of Singapore Singapore Singapore; ^2^ Antimicrobial Resistance Interdisciplinary Research Group Singapore‐MIT Alliance for Research and Technology Singapore Singapore; ^3^ NUS Environmental Research Institute National University of Singapore Singapore Singapore; ^4^ Monterey Bay Aquarium Research Institute Moss Landing California USA; ^5^ GEOMAR Helmholtz Centre for Ocean Research Ocean EcoSystems Biology Unit Kiel Germany; ^6^ Department of Biological Engineering Massachusetts Institute of Technology Cambridge Massachusetts USA

**Keywords:** flow cytometry sorting, modified Ludzack–Ettinger treatment, viral metagenomics, wastewater

## Abstract

Traditional or “bulk” viral enrichment and amplification methods used in viral metagenomics introduce unavoidable bias in viral diversity. This bias is due to shortcomings in existing viral enrichment methods and overshadowing by the more abundant viral populations. To reduce the complexity and improve the resolution of viral diversity, we developed a strategy coupling fluorescence‐activated cell sorting (FACS) with random amplification and compared this to bulk metagenomics. This strategy was validated on both influent and effluent samples from a municipal wastewater treatment plant using the Modified Ludzack–Ettinger (MLE) process as the treatment method. We found that DNA and RNA communities generated using bulk samples were mostly different from those derived following FACS for both treatments before and after MLE. Before MLE treatment, FACS identified five viral families and 512 viral annotated contigs. Up to 43% of mapped reads were not detected in bulk samples. Nucleo‐cytoplasmic large DNA viral families were enriched to a greater extent in the FACS‐coupled subpopulations compared with bulk samples. FACS‐coupled viromes captured a single‐contig viral genome associated with Anabaena phage, which was not observed in bulk samples or in FACS‐sorted samples after MLE. These short metagenomic reads, which were assembled into a high‐quality draft genome of 46 kbp, were found to be highly dominant in one of the pre‐MLE FACS annotated virome fractions (57.4%). Using bulk metagenomics, we identified that between Primary Settling Tank and Secondary Settling Tank viromes, *Virgaviridae*, *Astroviridae*, *Parvoviridae*, *Picobirnaviridae*, *Nodaviridae*, and *Iridoviridae* were susceptible to MLE treatment. In all, bulk and FACS‐coupled metagenomics are complementary approaches that enable a more thorough understanding of the community structure of DNA and RNA viruses in complex environmental samples, of which the latter is critical for increasing the sensitivity of detection of viral signatures that would otherwise be lost through bulk viral metagenomics.

## Highlights

- The practical protocol of applying fluorescence‐activated cell sorting (FACS) and metagenomics on DNA and RNA viromes.
- The first systematic analysis of viral metagenomics in a municipal wastewater treatment plant with Modified Ludzack–Ettinger (MLE) processes.
- Coupling FACS with metagenomics improved the detection and genome assembly of certain rare viral species.
- Bulk viral metagenomics and FACS‐coupled metagenomics delivered complementary results.

## Graphical abstract

This study elucidates the practical protocol of applying fluorescence‐activated cell sorting (FACS) and metagenomics on DNA and RNA viromes. This is the first systematic analysis of viral metagenomics in a municipal wastewater treatment plant with the Modified Ludzack–Ettinger (MLE) process. Coupling FACS with metagenomics improved the detection and genome assembly of certain rare viral species. In conclusion, bulk viral metagenomics and FACS‐coupled metagenomics delivered complementary results.

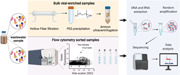

## INTRODUCTION

Viruses are the most common biological entities in the biosphere. Their abundance has been estimated at 10^7^−10^9^ virus‐like particles (VLPs)/ml in sediments, oceans, and soil [1, 2], and 10^9^−10^12^ VLPs/g feces in the human gut [3]. Although viruses fill important and broad niches in the environment, the knowledge of their ecology is limited due to the difficulties in virus isolation, culturing, and maintenance of host–virus systems [4, 5]. Furthermore, the lack of universal viral genetic markers, such as the 16S rRNA gene shared by all bacteria, also hinders the understanding of viral diversity, phylogeny, and taxonomy [6]. Advances in next‐generation sequencing platforms and bioinformatics jumpstarted a renewed interest in resolving viral communities that are previously undetermined (i.e., the so‐called “viral dark matter”) [7, 8]. For example, it has successfully been used to resolve viral community structures from specific stages (i.e., raw sewage, sewage sludge, methanogenic digesters, etc.) in the wastewater treatment plant (WWTP) [9, 10, 11, 12, 13, 14, 15, 16, 17, 18]. In sewage samples, DNA viromes are dominated by bacteriophages that likely infect enterobacteria or lactococci [9] while plant viruses, such as *Virgaviridae*, dominate RNA viromes [19, 20, 21, 22]. Human pathogenic and emerging zoonotic viruses, such as *Adenoviridae*, *Astroviridae*, *Picornaviridae*, *Calicviridae*, *Papillomaviridae*, and *Hepesviridae*, have also been detected in wastewater through metagenomics [10].

Understanding the dynamic changes in microbial diversity and composition of the wastewater treatment process is essential, particularly where effluents are intended for reuse. However, studies remain hampered by difficulties in virus enrichment, amplification, and low sensitivity and specificity of viral classification by current bioinformatics workflows [11, 12]. Typical viral enrichment approaches, such as physical size filtration (0.22 μm), aim to increase the virus fraction while avoiding bacterial contamination. Unfortunately, viral particles are very heterogeneous in virion size and shape. They can be as small as 20−30 nm for some viruses (*Astroviridae*, *Picornaviridae*, *Circoviridae*), 110−150 nm (*Coronaviridae*, *Herpesviridae*) in medium size [13], and up to 1‐µm virion size for nucleo‐cytoplasmic large DNA viruses (NCLDVs), commonly referred to giant viruses [14]. The latter, which has a genome size of 0.1–2.77 million bp, can be easily removed through 0.22‐μm filtration. Despite the filtering step with potential losses of giant viruses, the reconstruction of viral genomes is further hampered by unavoidable bacterial host contamination. In fact, bacteria with nonuniform morphologies could pass through 0.22‐μm pores [19]. Furthermore, wastewater samples contain a complex mixture of bacterial and viral species, including single‐stranded or double‐stranded RNA and DNA viruses. The majority of the studies in wastewater still focus on dsDNA viruses. RNA viromes, on the other hand, have received increased attention since most of the RNA viruses are human, plant, or insect pathogens and are of public health importance. The sequence‐independent single primer amplification (SISPA) approach has been used by studies focusing on either RNA [20] or DNA viruses [21] or both [23, 24, 25]. Thus far, it remains highly challenging to purify and isolate the entire viral population from complex water matrices.

Aside from the upstream viral enrichment process, there are several barriers in downstream bioinformatics analysis that limit viral community identification and characterization. High viral complexity and diversity in the environmental samples, particularly sewage samples, often lead to nonuniform sequencing depth resulting in loss of rare viral species signals and poor quality of viral genome recovery [26, 27, 28]. Recently, flow cytometry has been coupled with viral metagenomics in marine samples to discover tailed and very large viruses, which could be excluded in routine viral metagenomics [29, 30, 31]. Single virus sorting helped in identifying 44 abundant single‐amplified viral genomes, which were previously unidentified in the ocean [32]. Through physically fractionating viral assemblages, higher sequence coverage and greater assembly of viral sequences were obtained [33], and subsequently, taxonomy prediction accuracy was improved [34]. It was reported that 2–10 times sequence coverage was required for 60%–95% recovery of viral genomes [34]. All these findings indicate that flow cytometry could be a feasible tool to improve genome assembly and annotation or detect different viruses that are presumably in low abundance and thus complement results obtained by traditional viral metagenomics.

To overcome the challenges of genome assembly or annotation of viruses that are low in abundance, here we use fluorescence‐activated cell sorting (FACS) by flow cytometry to sort wastewater viromes into distinct FACS subpopulations. These methods are demonstrated on the Primary Settling Tank (PST) and Secondary Settling Tank (SST) effluent in the wastewater treatment stage of a municipal WWTP. Next‐generation sequencing using Illumina Hiseq 2500 platform was applied and bioinformatics analysis was then used to characterize the unsorted bulk virome and FACS subpopulations to identify viral signatures lost using bulk viral metagenomics. Using diversity metrics, we derived how various sample processing methodologies affected the species richness and compared pre‐ and post‐Modified Ludzack–Ettinger (MLE) samples to identify viral populations that were susceptible to MLE. In all, this study constitutes a descriptive study comparing FACS‐metagenomics with bulk viral metagenomics on pre‐ and post‐MLE samples from a Singaporean WWTP and demonstrates the utility of combining the two approaches to enhance virome coverage.

## RESULTS

### FACS, amplification, and sequencing statistics

One of the objectives of this study is to explore how RNA and DNA viromes derived from bulk metagenomics may be complemented by FACS‐coupled metagenomics. To capture both DNA and RNA viruses, we utilized two strategies. First, we used SYBR Gold to stain both DNA and RNA viruses in the FACS subpopulations instead of SYBR GREEN I, which preferentially binds to dsDNA and with low binding performance to ssDNA and RNA. Second, we performed the random amplification approach utilizing SISPA theory [35] to amplify the extracted nucleic acids in both the bulk virome and FACS subpopulations. The advantage of pooling both RNA and DNA viruses for coupled processing and sequencing is that it provides a fast and convenient approach.

**Figure 1:**
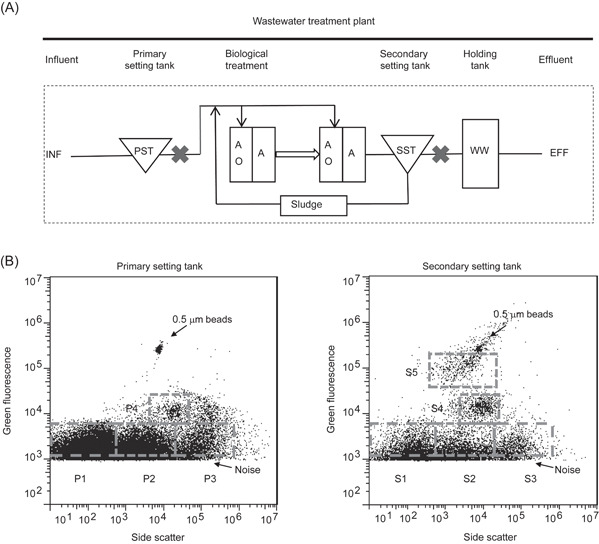
Sampling locations and FACS‐sorted subpopulations. (A) Schematics of the wastewater treatment plant in Singapore. Sampled points are denoted by “**Χ**.” Wastewater influent is channeled into the PST where organic and inorganic matter settles to the bottom and is removed as waste sludge. PST effluent is pumped into separate trains for biological treatment, a step‐feed MLE (anoxic‐oxic‐anoxic [AOA]) process before being fed into a Secondary Settling Tank (SST). Settled sludge from SST is pumped back into the initial MLE stage as an activated mixed liquor source for biological treatment [36]. (B) Subpopulations of P1–P4 (left panel) from PST and S1–S5 (right panel) from SST wastewater samples were sorted using flow cytometric sorting. Discrimination of groups was based on green fluorescence and side scatter signatures. EFF, wastewater effluent; FACS, fluorescence‐activated cell sorting; INF, wastewater influent; MLE, Modified Ludzack–Ettinger; PST, Primary Settling Tank; WW, wet well.

Wastewater samples were obtained from PST and SST. SST had been subjected to MLE within the WWTP. Following staining by SYBR Gold, each sample was sorted into four and five subpopulations, respectively (i.e., PST, 1–4; SST, 1–5), based on their fluorescence and side scatter (SSC) signals (Figure 1). After nucleic acids extraction, random amplification, and high‐throughput sequencing, 21.4 ± 2.71 million high‐quality reads were obtained from each library (17.4–25.6 million) and 52.6 ± 8.4% of high‐quality reads mapped back to the assembled contigs per library (43.0%–69.6%) on average (Table S2). The general bioinformatics workflow is depicted in Figure S1. We used a coassembly strategy to obtain a total of 43,582 contigs with the contig length N50 value of 1653bp from 11 samples. These contigs were annotated in parallel, comparing a database‐dependent approach (BLASTP against NCBI non‐redundant protein database and NCBI viral protein database, Megan lowest common ancestor (LCA) assignment, details in Methods) against a non database‐dependent algorithm VirFinder and a database‐dependent algorithm VirSorter. Within these, 4417 contigs were annotated as viruses in the NCBI database and 38 viral families were detected. In parallel, 5514 contigs were predicted to be of viral origin by applying VirFinder v1.1 (score ≥0.9 with *p* < 0.01) and VirSorter (Cat 1 and 2, virome decontamination mode). There was an overlap of 215 annotated contigs between VirFinder and VirSorter approaches (Figure S2A) of which VirFinder delivered a higher number of viral signal hits (VirFinder vs. VirSorter: 4880 vs. 849). Between NCBI BLASTP and VirFinder/VirSorter, 1395 annotated contigs were common to both of them, and 8536 contigs were unique (Figure S2B).

### DNA and RNA viruses detection in both bulk virome and FACS subpopulations

Following the contig assignment, we examined and compared the relative abundance of eukaryota‐, bacteria‐, archaea‐, and viral‐affiliated contigs between bulk viral metagenomes against those identified in FACS subpopulations. The relative abundance was calculated based on the mapped reads ratio of contigs assigned to a specific taxonomy. Bulk samples were prepared using traditional viral enrichment steps (i.e., polyethylene glycol [PEG] precipitation, chloroform purification, 0.22‐μm filtration, and Amicon centrifuge) to increase the viral signal‐to‐noise ratio. FACS samples, on the other hand, were subjected to FACS in the absence of prior viral enrichment to enable the capture of bacteria and microorganisms beyond viruses. As such, bulk metagenomes (enriched in viruses, PST, and SST) included a lower abundance of annotated bacteria, archaea, and eukaryota (31 ± 0.18%) compared with FACS subpopulations (68.3 ± 14%; Table S3). PST and SST had a higher percentage of mapped reads assigned to known sequences in the ViralRefSeq database (56.6% and 48.3%, respectively), while FACS subpopulations (P1–P4 and S1–S5), in the absence of traditional viral enrichment, had a lower proportion (2.1 ± 0.6%; Table S3).

Following that, we identified and annotated 2673 DNA and 118 RNA viruses from both bulk and FACS subpopulations. Fifty‐sixth out of 118 contigs were affiliated to plant‐related viruses belonging to *Virgaviridae* (best hits to Cucumber green mottle mosaic virus, Tobacco mild green mosaic virus, and Pepper mild mottle virus), *Tombusviridae* (best hits to Melon necrotic spot virus and Maize chlorotic mottle virus), and *Alphaflexiviridae* (Potexvirus, best hits to Schlumbergera virus X, Pitaya virus X, Cactus virus, and Zygocactus virus X). Contigs assigned to human‐related viruses (human adenovirus and human astrovirus) and crAssphage were recovered in PST and SST but not detected in FACS subpopulations (Table S7 and Figures S10 and S11).

### The relative abundance of NCLDVs, certain human viral families, and crAssphage decreased after MLE treatment

**Figure 2:**
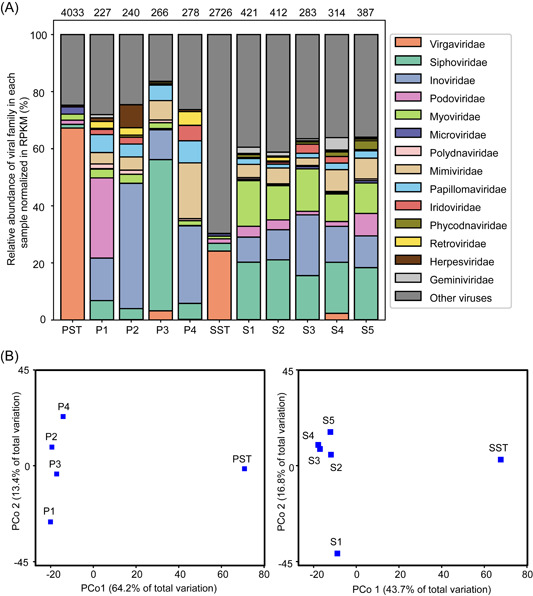
Viromes distribution in both bulk and FACS subpopulations. (A) Taxonomic percentage of annotated sequences normalized in RPKM across 11 libraries. The top 14 most abundant viral families across 11 libraries were plotted and the remaining viral families were represented as “Other viral families.” Unassigned contigs at the family level were represented as “Other viruses.” For each subpopulation, approximately 2 million particles were sorted out and subsequently analyzed. The number of virus annotated contigs through the Megan LCA assignment approach in each sample was indicated above the data columns. (B) Principal coordinate analysis (PCoA) based on contig‐level Bray–Curtis dissimilarity derived from taxonomy‐affiliated contigs of PST and sorted subpopulations (P1–P4; left panel) and SST and sorted subpopulations (S1–S5; right panel). The display is based on sample scores on the primary axis and secondary axis. Left panel—PCoA1, 64.2% variance explained; PCoA2, 13.4% variance explained. Right panel—PCoA1, 43.7% variance explained; PCoA2, 16.8% variance explained. FACS, fluorescence‐activated cell sorting; LCA, lowest common ancestor; PST, Primary Settling Tank; RPKM, reads per kilobase of contig per million mapped reads; SST, Secondary Settling Tank.

Next, we focused on the viral families within annotated viromes in bulk viral metagenomes. The relative abundance of viral families was quantified based on the reads per kilobase of contig per million mapped reads (RPKM) value. Of the annotated PST viromes, 67.2% of reads were assigned to contigs classified as *Virgaviridae*, 2.5% *Microviridae*, 2.1% *Myoviridae*, 1.5% *Podoviridae*, and 1.2% *Siphoviridae* (Figure 2). The high prevalence of *Virgaviridae* (67.2%) is similar to the findings of a WWTP RNA virome (>80% of *Virgaviridae*) in Saudi Arabia [37], untreated sewage viromes (57% *Virgaviridae*) in Nepal [23], and sewage bulk RNA viromes (the most abundant viruses were *Virgaviridae*) in Southern California [38, 39]. The SST virome consisted of 24.1% of *Virgaviridae*, followed by *Podoviridae* (1.6%), *Siphoviridae* (2.7%), and *Myoviridae* (1.0%; Figure 2). Interestingly, the SST virome consisted of approximately 68.4% of “other viruses” (i.e., unassigned contigs at the family level), of which 16.1% is Bufivirus UC1 (Figure S3), a virus detected from San Francisco wastewater [40].

Comparing the relative abundance of viral families between PST and SST effluent, we found that 25 viral families decreased during the MLE process in the WWTP. The percentage of *Virgaviridae* decreased from 67.2% to 24.1% while *Microviridae* and *Myoviridae* dropped from 2.1% to 0.9% and from 1.5% to 1.0%, respectively. NCLDVs, an order of giant viruses, were detected in this study (e.g., *Mimiviridae*, *Phycodnaviridae*, *Poxiviridae*, *Iridoviridae*, and *Marseilleviridae*). The crAssphage (unclassified *Podoviridae*), which has been identified as prevalent in human fecal samples [41], was discovered in PST at a percentage of 0.17% and decreased dramatically to 0.00067% in SST (Figure S3). The relative abundance of *Adenoviridae* and *Astroviridae* decreased dramatically (Figure S10). Specifically, all the contigs in *Adenoviridae* and *Astroviridae* were affiliated to human adenovirus and human astroviruses (98%–99% identity and 99% query coverage, Table S7). The relative abundance of *Podoviridae*, *Siphoviridae*, *Circoviridae*, *Disctroviridae, Herpesviridae*, *Retroviridae*, and *Caliciviridae* did not decrease after the MLE process, suggesting MLE process did not effectively remove these viruses. For taxonomy‐affiliated viral contigs, the α‐diversity estimates for PST and SST were 2375 and 998 species with Shannon indexes of 5.3 and 4.0 (Figures S4 and S5). The lower biodiversity in SST indicates that the MLE process in the WWTP removed the majority of viral species.

### Virome differences between bulk and FACS‐sorted samples, and among FACS subpopulations

Next, we measured the extent to which viral community structure differed between bulk viral samples and FACS subpopulations. Viral community structure was divided into two main clusters along the first and second axes of a principal coordinate analysis (PCoA): one represented by PST or SST sample and another consisting of their subpopulations (Figure 2B) and significant differences were observed (PERMANOVA statistics, *p* = 0.013, pseudo‐*F* = 5.3109, Table S8). A similar trend was observed in the VirFinder and VirSorter contigs (Figure S6).

Compared with 2375 and 998 species detected in PST and SST, sorted subpopulations had lower numbers in P1–P4 (170–277 species) and S1–S5 (181–337 species; Figure S4, *p* < 0.05, Kruskal–Wallis test). As expected, FACS fractionated the whole community and hence, the species richness under each sorted category decreased when compared with the bulk virome. Interestingly, P1–P4 had a relatively lower α‐diversity than S1–S5 (3.4–3.7 vs. 4.8–6.8, *p* < 0.05, two‐tailed Mann–Whitney test), indicating that P1–P4 was dominated (>40%) by certain contigs.

Hence, we sought to determine what dominated P1–P4 viromes. P1, particles with the lowest SSC and green fluorescence signals (Figure 1B), were dominated by unclassified *Podoviridae* (28.1%) and *Inoviridae* (14.9%). Further examination of the top most abundant contig under *Podoviridae* in P1 showed that this contig had hypothetical genes annotated as Anabaena phage A‐4L at amino acid level (1E‐32, 26% identity, 29% query coverage across a length of 10,136 bp, BLASTX to NCBI nr), although there were no hits to the current NCBI nt database at a nucleotide level (Table S5). While this contig may be too distant to be annotated in the current updated NCBI nt database, it shared functional genes with Anabaena phage, which was originally isolated from sewage settling ponds [42]. P2, particles with intermediate SSC and lowest green fluorescence signals in flow cytometry, were dominated by *Inoviridae* (43.9%). The top contigs in P2 were fully affiliated (100% nucleotide identity and 100% query coverage across a length of 3427 bp) to *Inoviridae*, Enterobacteria phage M13, a 6.4‐kbp ssDNA phage (Table S5). This result is consistent with a previous study showing that around 20% of the known bacteriophage reads infect enterobacteria in raw sewage [9]. P3, particles with the large SSC and lowest green fluorescence signals in FCM, were dominated by *Siphoviridae* (53.0%) and *Inoviridae* (10.4%). The top contig in P3 was affiliated to *Siphoviridae*, with the gene annotated as DNA ligase associated most closely to *Pseudomonas* phage (2E‐10, 56% identity, 13% query coverage). P4, particles with intermediate SSC and intermediate green fluorescence signals in FCM, were dominated by *Inoviridae* (27.1%), *Mimiviridae* (19.6%), *Papillomaviridae* (7.7%), and *Iridoviridae* (5.4%). From P1 to P4, the percentage of *Mimiviridae* was found to increase from 4% to 19.6% with increasing SSC and green fluorescence signals and 1.8% to 5.4% for *Iridoviridae*, respectively.

In contrast to viral distributions of P1–P4 (the mean abundance standard deviation [STD] of the top 14 most abundant viral families: 5.5%), the relative abundance of viral families in S1–S5 was more evenly distributed (the mean abundance STD: 1.6%), except for *Inoviridae*, which accounted for 21.3% in S3 with an abundance STD of 4.9%. The viral family evenness is in accordance with the higher Shannon index observed (4.8–6.8) compared with P1–P4 (3.4–3.7; Figure S4). As with P1–P4, the same trend was also observed for S1–S5 in that *Mimiviridae* increased from 4.6% to 7.6% with increasing SSC and green fluorescence signals. Another giant viral family, *Phycodnaviridae* increased from 0.4% to 3.0% from S1 to S5. The percentage of *Virgaviridae* decreased from 67.2% in the PST total sample to 0.01%–3.2% of the sorted subpopulations P1–P4 and from 24.1% in the SST total sample to 0.1%–2.3% in the sorted subpopulations S1–S5.

### More nucleo‐cytoplasmic large DNA viral families were detected in the FACS‐sorted subpopulations compared with bulk samples

**Figure 3:**
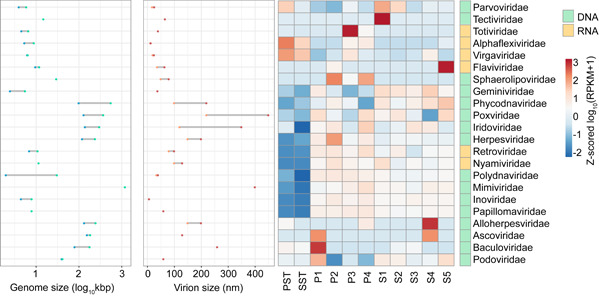
Genome size (log_10_ transformed kbp) and virion size (nm) and Normalized abundances (log_10_(RPKM + 1) transformed counts per kilobase contig per million reads) of viral families identified in all the samples (DNA viral families are denoted in green while RNA viral families are denoted in yellow in sidebar annotation). Rows are centered; unit variance scaling is applied to rows. Rows are clustered using correlation distance and average linkage: 22 rows and 11 columns. Virion sizes were obtained from the ViralZone website: www.expasy/org/viralzone, Swiss Institute of Bioinformatics. Viral families were selected based on two criteria that (1) they are abundant (log_10_(RPKM + 1) > 1.22) and (2) they have a contrasting difference between each sample (STD of log_10_(RPKM + 1) > 1.32)). The threshold was calculated as follows: for each viral family, the log_10_(RPKM + 1) value across 11 samples was averaged, and the medium number of the averaged values was calculated from 38 viral families. PST, Primary Settling Tank; RPKM, reads per kilobase of contig per million mapped reads; SST, Secondary Settling Tank; STD, standard deviation.

Although bulk virome retained a higher abundance of viral signals, many reports indicated that NCLDVs have been lost during the enrichment procedure. Here we examined the relative abundance of NCLDVs in both FACS subpopulations and bulk viromes. At the family level, most of the NCLDVs were abundant in the sorted subpopulations compared with PST and SST, including *Phycodnaviridae*, *Poxviridae*, *Iridoviridae*, *Nyamiviridae*, and *Mimiviridae* (Figure 3). Five families (*Tectiviridae*, *Sphaerolipoviridae*, *Ascoviridae*, *Nyamiviridae*, and *Flaviviridae*) were undetected in both PST and SST but identified in their sorted subpopulation samples (Figure S7). *Papillomaviridae* and *Alloherpesviridae*, which were undetected in SST, were identified in S1–S5 (Figure S7). *Ascoviridae* (130 nm in diameter and 200–400 nm in length) and *Nyamiviridae* (100–130 nm in diameter) belonged to NCLDVs. These results are expected as bulk viral metagenomics will undersample giant viruses through 0.22‐μm size‐exclusion during the filtration process. *Tectiviridae* (66 nm with apical spikes of 20 nm) was detected in S1, *Flaviviridae* (40–60 nm in diameter) was detected in S5, and *Totiviridae* (40 nm in diameter) was detected in P3 (Table S4). As particles in S5 and P3 were characterized by larger scatter size, the detection of *Totiviridae* and *Flaviviridae* within these subpopulations may arise from the aggregation of viral particles due to insufficient filtration and centrifugation [43]. Interestingly, compared with the PST and SST, *Virgaviridae* and *Alphaflexiviridae* decreased in abundance after sorting, which could be attributed to their rod‐shaped structure, creating difficulty in sorting. Furthermore, RNA viruses are fragile and could have low sorting efficiency (Figure 3).

### Target‐sorted viromes captured contigs associated with Anabaena phage, which would otherwise be lost in the bulk viral metagenome

**Figure 4:**
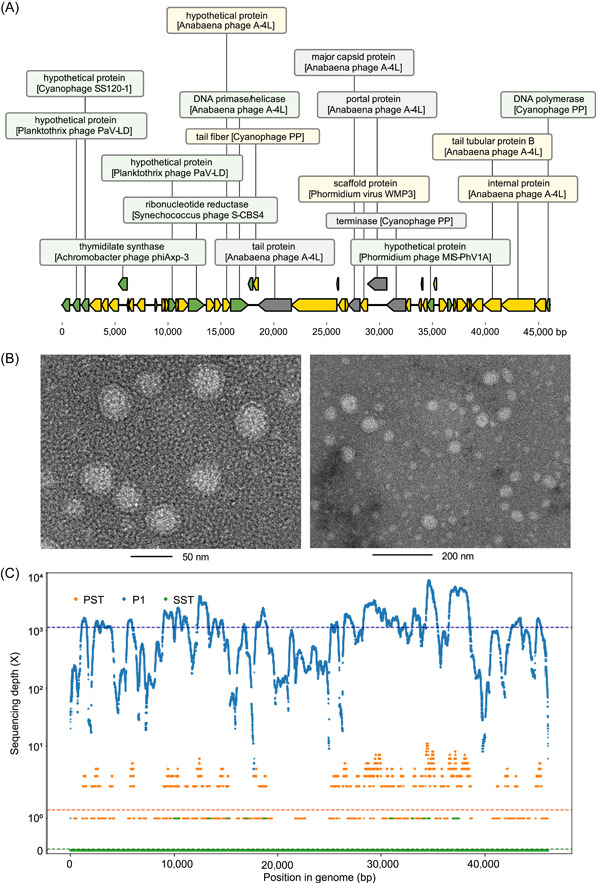
P1 revealed highly enriched phages (P1_MAG1) that were not present in the bulk viral metagenome (PST and SST). (A) The genome annotation plot of P1_MAG1 was generated with DnaFeaturesViewer [44]. Contigs ≥5 kbp were piped through VirSorter 2 and DRAMv for viral identification and annotation. Multiple hits matched to Anabaena phage A‐4L. Green denotes VirSorter category 1, yellow denotes VirSorter category 2, gray denotes signals that fall into other VirSorter categories or no hits. (B) Transmission electron micrographs of the most prevalent phages in P1 reveal their morphological characteristics. (C) Fragment recruitment of reads from P1‐sorted, PST, and SST bulk metagenomes onto the single‐contig viral genome P1_MAG1. Reads matching ≥70 bp in length and ≥70% nucleotide identity were used as cutoffs. In the P1‐sorted metagenome, an average of 1136 sequencing depth (X) of P1_MAG1 was observed, but a low sequencing depth (1.26X and 0.04X) of P1_MAG1 was observed in the PST and SST bulk metagenomes, as represented by horizontal dashed lines. PST, Primary Settling Tank; SST, Secondary Settling Tank.

Challenges in viral metagenomics hinder the recovery of certain viruses by bulk viral enrichment procedures and downstream bioinformatics analysis. It is hypothesized that target‐sorted viromes will capture viruses that would otherwise be lost in normal viral metagenomics. Hence, we extracted and focused on contigs that showed no reads mapped in PST and SST. To capture viral‐affiliated contigs at high resolution, the RPKM matrix of viral‐affiliated contigs was summed up to their lowest taxonomy level and clustered across sorted subpopulations (Figure S8). Most of the virus sequences had close hits to Caudovirales, phages that infect *E. coli*, *Pseudomonas*, *Erwinia*, *Bacillus*, *Shigella*, *Vibrio*, *Clostridium*, and *Synechococcus* (Figure S8). Sequences with the best blast hit to other viruses infecting hosts, such as ameba, algae, birds, fish, invertebrates, and ruminants, were also detected. It is also worth noting that the P1 top most abundant contig k141_468300 (10,136 bp, best hit to Anabaena phage A‐4L, 26% identity, 29% query coverage, 94,669 mapped reads, Table S5) accounted for more than 20% in P1 annotated virome, but had fewer reads mapped to S1–S5, PST, and SST (<10 mapped reads; Table S5 and Figure S9). MetaSPAdes was used as an alternative assembly technique to produce a more complete viral genome.   Subsequenced P1 assembled with metaSPAdes resulted in a 46,094 bp genome with 49 open reading frames (ORFs), which represents the 4.5‐fold improvement in viral genome breadth recovery compared with MEGAHIT (10,136 bp; Figure 4A). We named this contig as P1_MAG1 and it was identified as category 1 and dsDNA phage (max score 0.927) in VirSorter2. Anabaena phage signals were annotated on the majority of its ORFs. The virion population in P1‐subsorted samples was roughly 50 nm, according to the transmission electron microscope (TEM) examination (Figure 4B). While P1 had a larger sequencing depth of 1136X, the low sequencing depth in PST (1.2X) and SST (0.04X) made it challenging to assemble and identify this draft genome at the bulk virome level (Figure 4B). As a result, sorting into subpopulations allows for the discovery and dynamics of this draft genome at the FACS‐metagenome level rather than the bulk virome level. We also performed rarefaction curve analysis to see if our sequencing depth was enough to cover potential low abundant species in bulk virome. The near plateauing rarefaction curve indicated that our sequencing depth was able to sufficiently cover the rare species for PST and SST samples (Figure S5). Following that, deep sequencing depth may not always recover rare contigs by bulk viral metagenomics, even though reads recruitment analysis was performed using the available reference genomes. Similar findings showed that genome reconstruction coverage did not show significant improvement even after increasing sequencing depth by 10‐folds [45]. As a result, coupling the FACS‐metagenomic approach with viral metagenomics leads us to a new way of improving genome reconstruction in complex environmental matrices.

## DISCUSSION

Recently, FACS has been successfully applied to characterize viral genomic and/or metagenomics to bridge the knowledge gaps between virology culture and viral metagenomics in different ecosystems, particularly marine waters and human gut microbiome [29, 30, 31, 32, 46, 47]. In a previous study, researchers were able to sort and sequence dsDNA viruses from up to three subpopulations (5000 particles each subpopulation) from 1‐ml marine water to discover giant viruses and other relevant uncultured viruses [30]. In our study, moving beyond that, by using four‐ and five‐way sorting and staining particles with SYBR Gold dye followed by random amplification of DNA and RNA, we attained 2 million particles in each subpopulation simultaneously in municipal wastewater samples. High numbers of sorted particles would reflect improved resolution for deeper characterization of virome distributions in complex wastewater samples, together with bulk virome, revealing a more precise and high‐throughput ecological survey, particularly for capturing rare species.

Marked differences in virome composition were observed between PST, SST, and FACS subpopulation samples with several possible explanations. First, the bulk viral enrichment approach used for PST and SST samples is more likely to remove nonviral fractions and retain higher viral fractions than the FACS‐metagenomics samples. Traditional virome enrichment involves background depletion of sequence signals, which inevitably introduce biased virome coverage, especially for the loss of giant viruses. Furthermore, the complexity of the environmental DNA will inhibit sequence amplification, resulting in imprecise virome identification and quantification. Second, differences in viral morphology, nucleic acid types, and virion size can affect sorting efficiency via size fractionation and fluorescence signals. For example, possibly due to their rod‐shaped morphology, *Virgaviridae* (ssRNA, 300 nm in length and 20–25 nm in diameter) decreased dramatically from PST (>60%) to FACS subpopulations, which, in turn, changes the virion community distribution patterns. The case applies to *Alphaflexiviridae* (ssRNA, 470–800 nm in length and 12–13 nm in diameter), which were more abundant in bulk viral metagenomes than in FACS subpopulations. These results suggest that viral three‐dimensional property serves as determining factors, shaping the abundance distribution.

Metagenomic assembly is particularly challenging for virome data, often resulting in fragmented assemblies and poor recovery of viral community members [48]. Although different algorithms and genome assemblers exist, in our study, using SPAdes with the “meta” option, we were able to successfully de novo assemble a high‐quality draft Anabaena phage genome in FACS subpopulations, which were not detected in bulk viromes. Sorting particles into subpopulations with similar virion size, genome size, or amount of nucleic acid, could reduce viral species richness in complex environmental communities, increase sequence coverage, and improve genome assembly. The average genome depth (1136X) of rare viruses such as Anabaena phages increased in some subpopulations by around 900–28,400‐folds compared with complex PST and SST samples (1.26X and 0.04X), thus enabling a near‐complete genome assembly. Through physically fractionating viral assemblages, high coverage will be obtained, enabling greater assembly of viral sequences [33], subsequently improving taxonomy prediction accuracy as reported. Combining bulk virome and FACS‐coupled metagenomics will provide wider coverage of particles. However, likewise in this study, an avoidable challenge exists in all assemblers, which is that strain heterogeneity within populations hinders viral assembly [49].

Bulk metagenomes, as a culture‐independent method, have been applied to investigate the role of viruses in the biological and ecological processes of wastewater treatment [50], while low abundant viral signals may be undetected or detected with uneven depth. For targeted and known viral targets, some researchers use targeted sequencing to provide high depth of epidemiologically informative regions of the genome [51], whereas for unknown viral targets, aside from deep sequencing technology, some other upstream processing technology should be considered, as described in this study, using FACS technology in conjunction with metagenomics to enable a deep understanding of the community. In our study, when comparing PST and SST at the total virome level, the relative abundance of different viral families demonstrates that *Virgaviridae*, *Astroviridae*, *Parvoviridae*, *Picobirnaviridae*, *Nodaviridae*, and *Iridoviridae* are more sensitive to MLE treatment in WWTP than *Podoviridae*, *Siphoviridae*, *Circoviridae*, *Disctroviridae, Herpesviridae*, *Retroviridae*, and *Caliciviridae*. In the MLE process, viral particles may attach to organic particulates or be absorbed into bioflocs through bioflocculation [52]. The bioflocs and organic particulates sediment in the settling process led to the reduction of viruses in the effluent. Physiochemical differences between viruses could have resulted in some viral families being removed to greater extents than others. Despite this not being the intended purpose and design, this process is unavoidable and presents as an unintended and yet purposeful result of MLE in tandem with the settling process [53, 54]. At the subdivided virome level using FACS, the most abundant viral contigs affiliated to cyanophages (Anabaena phages A‐4L) detected in P1 had low abundance in S1–S5, indicating the potential removal of small cyanophages in the MLE process. As such, bulk viral metagenomics and FACS metagenomics delivered complementary results, facilitating the understanding of the inner mechanisms of ecosystem dynamics and wastewater treatment process at a higher resolution level, especially understanding the viral structure variation with different virion size and green fluorescence signals. The FACS approach, coupled with the random amplification protocol used in this study, provides a potentially new and powerful method to selectively enrich and genetically characterize rare and potential novel viruses.

## LIMITATIONS

While we have demonstrated and described the utility of FACS in improving the resolution of community structure, there are some limitations in our study. Genomic DNA and RNA in the samples were extracted using the QIAmp Viral RNA mini kit. While the recovery rate of DNA viruses was not evaluated in this study, the QIAamp Viral RNA Mini Kit has been commonly used to recover both DNA and RNA viral pathogens [55]. Further, an earlier study reported that the QIAamp RNA extraction kit performed well among four different commercial kits in recovering both DNA virus (Adenovirus) and RNA viruses (influenza virus A [enveloped single‐stranded, segmented RNA virus], human coronavirus OC43 [enveloped positive‐stranded RNA virus], and human metapneumovirus [enveloped negative‐stranded RNA virus]) in respiratory clinical samples [56]. Nonetheless, further studies are needed to investigate the extraction efficiency of a broader range of DNA and RNA viruses to guide the choice of extraction kits. Moreover, FACS has certain limitations. In this study, RPKM of *Virgaviridae* and *Alphaflexiviridae* decreased after sorting, indicating that FACS might have a bias against the virion structure and nucleic acid type since these two RNA viral families have typical rod‐shaped structures. Further research should be conducted to assess and optimize the specificity of FACS. As mentioned earlier, contigs affiliated to human astrovirus, adenovirus, and crAssphage were recovered in PST (Table S7 and Figures S10 and S11); however, they were not detected in subpopulations. A possible reason could be that their small morphological size (MS2, 27 nm; Qbeta, 28 nm; Astrovirus, 33 nm) does not produce a strong SYBR Gold signal. Therefore, it may be impossible to differentiate this small ssRNA genome from the background noise [29]. It is worthwhile noting that *Microviridae* (30 nm, icosahedral) were detected in subpopulations (24 contigs in P1–P4 and S1–S5), although the majority of contigs were mapped to bulk viral samples (4418 contigs in PST and SST). Similarly, one contig affiliated to *Circoviridae* (17 nm, icosahedral) was detected in subpopulations with (P1–P4 and S1–S5), while 39 contigs were mapped to bulk viral samples. Recent studies indicate the feasibility to sort 50–60 nm diameter lambda phages through SSC [46]. However, the absence of Adenovirus (100 nm) and crAssphage (76.5 nm head size) in subpopulations after sorting warrants special attention and requires further investigation. One possible explanation could be that random amplification applied without 0.22‐µm size filtration in sorted subpopulations decreases the viral signal‐to‐noise ratio. Hence, in future studies, we recommend optimizing FACS on the basis of the virion structure, virion size, single‐ or double‐stranded viruses, and viral aggregation. Some straightforward and efficient steps, such as DNase treatment and size filtration, could be used to improve the efficacy of viral amplification before random amplification. Powerful nucleic acid staining dyes could be developed to improve the staining sensitivity for detecting particularly small virion sizes, especially in clinical settings. Furthermore, a larger sample size for improving statistical robustness and process controls, including influent and effluent samples without any prior viral enrichment treatment, is recommended for optimization of the FACS methodology and investigation of the viral taxonomy and functions in the systems.

## CONCLUSION

Viruses play an important role in biogeochemical cycles and in shaping microbial ecosystems. Evaluating the patterns and shifts in viral population dynamics using high‐resolution methods, together with metagenomics sequencing and stringent viral identification criteria, provide improved detection of viruses, especially human‐related viruses, which are major threats to public health. Here using PST and SST samples, we demonstrate the utility of combining FACS and metagenomics at providing a more thorough understanding of viromes in the pre‐ and post‐MLE stages of a municipal WWTP. We compared bulk viral metagenomics to FACS‐coupled metagenomics and found that the latter yielded a set of viromes that were distinctly different from those derived from bulk samples. Notably, more NCLDVs and Anabaena phages were identified with FACS but not with bulk viral metagenomics. In fact, FACS enabled the recovery of a high‐quality single‐contig viral genome of 46 kbp of the latter. Further, we compared bulk pre‐ and post‐MLE samples, and identified viral populations, in particular human viruses and crAssphage, which were susceptible to MLE and others (*Podoviridae*, *Siphoviridae*, *Circoviridae*, *Disctroviridae*, *Herpesviridae*, *Retroviridae*, and *Caliciviridae*) that were not. Overall, this paper has demonstrated how FACS‐coupled metagenomics could complement bulk viral metagenomics by improving resolution of viral structure variations in complex environmental matrices.

## METHODS

### Wastewater sampling

Two wastewater samples were each collected from PST and SST effluents with sampling volumes of 5 L and 60 L, respectively. Sampling was conducted on June 7, 2017 at a WWTP in Singapore and transported to the laboratory within 2 h in two autoclaved sterile containers. The schematic plot of the WWTP system and the function of PST and SST is detailed in Figure 1A.

### Bulk viral enrichment and purification of PST and SST wastewater samples

Primary and secondary concentration was performed for viral enrichment and purification in our study. In the primary concentration process, the 5 L PST and 60 L SST samples were concentrated immediately using a hollow fiber filtration unit (Hemoflow Fresenius HF 80S) with blocking (0.1 g of sodium pyrophosphate [NaPP; Sigma‐Aldrich] in 1 L of nanopure water) and elution buffer (0.1 g of NaPP, 5 ml of Tween 80 [Sigma‐Aldrich] and 20 μl of Antiform [Sigma‐Aldrich] in 1 L of nanopure water) to a final volume of 400 ml primary concentrate [24, 55]. In the secondary concentration process, 200 ml of primary concentrate wastewater was further concentrated using PEG precipitation (Sigma‐Aldrich), chloroform (Sigma‐Aldrich) treatment, 0.22‐μm sterile syringe size filtration (Sartorius), and Amicon Ultra‐15 centrifugal filtration (Merck Millipore; Supporting Information A) following our previous protocol [24]. After concentration, DNase treatment was performed using the RQ1 RNase‐free DNase kit (Promega) before nucleic acid extraction.

### Fluorescence‐activated cell sorting

One milliliter of raw wastewater subsamples from PST and SST was prefiltered using a filtering mesh (pore size, 60 µm; Sefar) to remove the large particles. The filtrates were fixed with 0.5% glutaraldehyde (Sigma‐Aldrich; final concentrations) [57, 58] for 2 h at room temperature, then another 15 min at 4°C, snap‐frozen in liquid nitrogen for 5 min, and finally stored at −80°C until sorting [30]. Fixed wastewater samples were thawed on ice and diluted 100‐fold with trisaminomethane–ethylenediaminetetraacetic acid buffer (pH 8.0, Biotechnology Grade). The diluted wastewater samples were treated with DNase (working concentration of 0.1 U/μl, Thermo Fisher Scientific) at 37°C for 30 min to remove the remaining free DNA and stained with SYBR Gold (1× final concentration, Thermo Fisher Scientific) for 10 min at 80°C in the dark before cooling for 5 min at room temperature as described previously [59]. The sorting of viral subpopulations was performed by a MoFlo Astrios EQ flow cytometer (Beckman Coulter). The wastewater samples were analyzed and sorted at 25 psi, and the viral subpopulations were discriminated based on SSC and green fluorescence signals (Excitation, 488 nm; Emission, 513 nm) with a threshold set at 10^3^ on fluorescein isothiocyanate (FITC). The 0.5‐µm calibration beads (Thermo Fisher Scientific) were used as the positive control in the experiment. On the basis of the SSC and FITC signal, four‐ and five‐way purity mode sorting were applied for four subpopulations from the PST and five subpopulations from the SST samples, respectively (abort rate <10%). Approximately 2 million particles of each subpopulation were sorted into 50 ml tubes for viral genome extraction and sequencing. All the sorted fractions were reanalyzed under the same condition, and the purity of each subpopulation must be >95% (sample purity [%] = particles still within the subpopulation/all particles × 100) [60].

Before the application for the samples in the current study, the FACS method was verified with an effluent sample collected from a local WWTP and a locally isolated cyanophage from the Singapore Serangoon Reservoir, PA‐SR01 [61]. Results showed the method had relatively high precision, with a relative STD ranging from 4.0% to 11.5% for the five subpopulations from the effluent sample and 3.6% for the cyanophage (Table S1).

### Extraction, random amplification, and sequencing of nucleic acids from sorted particles

PST, SST viral‐enriched samples, and nine FACS subpopulations (P1–P4 sorted from untreated PST samples and S1–S5 sorted from untreated SST samples) were lysed, and genomic DNA and RNA were extracted using the QIAmp Viral RNA mini kit (Qiagen) [62]. The extracted RNA was reverse‐transcribed to cDNA, and both DNA and cDNA were amplified following a previous random amplification protocol with few modifications [63, 64]. Briefly, random amplification includes reverse transcriptase (RT), the second synthesis, and polymerase chain reaction (PCR) amplification steps. During the RT step, 5 μl extracted RNA was mixed with 1 μl of 40 pmol/μl primer A (5′‐GTT TCC CAG TCA CGA TAN NNN NNN NN) and 4 μl of diethyl pyrocarbonate (DEPC)‐treated water (Invitrogen) and incubated at 65°C for 5 min and 4°C for 5 min. After that, the mixture was added with a 10‐μl mastermix consisting of 4 μl 5× SuperScript III RT buffer (Invitrogen), 1 μl 10 mM deoxynucleoside triphosphate (Promega), 1 μl RNAseOUT Recombinant Ribonuclease Inhibitor (Invitrogen), 0.5 μl DEPC‐treated water, 1.5 μl 0.1 M dithiothreitol (Invitrogen), and 2 μl SuperScript III RT (Invitrogen). The mixture was then incubated at 42°C for 60 min. Second‐strand DNA synthesis was performed with Sequenase (Affymetrix). Primer B (5′‐GTT TCC CAG TCA CGA TA) was added to amplify the 6 μl randomly primed cDNA for 40 cycles of PCR by using the following thermal setting: 94°C for 30 s, 40°C for 30 s, 50°C at 30 s, and 72°C at 60 s (Supporting Information B). To ensure there was no contamination during the nucleic acids extraction and random amplification steps, a negative control was run on 1% agarose gel to confirm that no DNA signal was detected in the negative control lane. The amplified materials were purified using Wizard SV gel and PCR clean‐up system (Promega) and quantified using a Qubit 3.0 fluorometer (Thermo Fisher Scientific) to achieve the sequencing quality standard.

A total of 11 samples (exclusive of the negative control sample) were sequenced at the Singapore Centre on Environmental Life Sciences Engineering. The Illumina TruSeq Nano DNA Library kit was used to construct libraries. The sequencing libraries with a corresponding insert size and adapters were prepared according to the previously described methods [65]. The libraries were then pooled and sequenced in two lanes with equimolar concentrations (except the negative control library) on an Illumina Hiseq 2500 sequencer in rapid mode at a final concentration of 10 pM and a read‐length of 250 bp paired‐end (V2 sequencing reagents).

### Processing and quantification of viromes

The sequences were quality‐filtered using BBtools (v38.22, detailed information in https://jgi.doe.gov/data-and-tools/bbtools/) to trim adapters, low‐quality reads, and “Primer B” sequences, which were used in the random amplification process and Phix reads. All metagenomic reads generated from bulk enrichment and FACS subpopulations were then coassembled using MEGAHIT (v1.1.2) with a minimum contig length of 1000 bp [66]. ORFs were extracted using Prodigal (2.6.3, meta mode) [67]. Bowtie2 (v2.2.6) was applied to map reads to contigs using default parameters [68]. A Python script was used to normalize the contig length and metagenome size to estimate the RPKM as stated before [24].

### Taxonomy assignment for viromes

Predicted ORFs were first subjected to BLASTP searches using Diamond (v0.8.22.84) against the NCBI non‐redundant protein databases (updated in December 2017) with default parameters except *E*‐value cutoff at 1E‐5 [69]. Megan 6 was used to assign taxonomy through the LCA algorithm with a threshold at 1E‐5 and bit score at 50 [70]. Generally, the LCA algorithm assigns reads or contigs to taxa reflecting the level of conservation of the sequence [71]. In some FACS subpopulations, particularly those with larger scatter sizes, we found frequent contamination of nonviral signatures (e.g., bacteria and eukaryotes). Hence, an in silico approach was applied to the first filter out contigs affiliated to bacteria, eukaryotes, and archaea (BLASTP queried against NCBI nr database, 1E‐5, Megan LCA taxonomy assignment). The remaining contigs were subjected to BLASTP searches against NCBI Viral RefSeq databases (updated in December 2017). In parallel, all the contigs assembled from MEGAHIT (>1 kb) were also annotated with VirFinder (V1.0.3, *p* < 0.01) and VirSorter (v1.0.3, Cat 1 and 2) following parameter settings indicated previously [72, 73, 74]. Additionally, for top abundant contigs (threshold setting: >3% of mapped reads to known viral sequences) in each FACS subpopulation, contig sequences were subjected to BLASTN and BLASTX searches against the NCBI nt and the NCBI nr database for nucleotide and protein annotation with *E*‐value cut off as 1E‐5. To annotate human‐related contigs, all the assembled contigs were subjected to BLASTN searches against the human viral pathogens database (https://www.ncbi.nlm.nih.gov/genomes/GenomesGroup.cgi, with the filtered keyword “human” by host group, downloaded in May 2020, with 12,181 complete viral RefSeq genotypes). The cutoff was set at 60% query coverage and 75% BLASTN identity and annotation results were manually curated to remove non human‐related viruses.

### Viral α‐diversity and β‐diversity

α‐Diversity and rarefaction curves were calculated in MacQIIME (version 1.9.1) using the rarefied absolute reads matrix (the nonnormalized matrix of reads mapped to each contig per sample) [75]. To calculate α‐diversity metrics (i.e., “Observed species” and “Shannon diversity index”) associated with taxonomy‐annotated viruses [76], the corresponding contigs were selected and the absolute reads matrix was rarefied to 124,811 reads (the smallest sample size) per sample. In parallel, to calculate α‐diversity associated with VirFinder and VirSorter affiliated viruses, the corresponding contigs were chosen from the rarefied matrix and the absolute reads matrix was rarefied to 426,969 reads (the smallest sample size) per sample.

PCoA was performed in PRIMERv7 based on Bray–Curtis similarity distance [77]. RPKM of the taxonomy‐affiliated contigs and of the VirFinder and VirSorter viruses were transformed using the log_10_(*x* + 1) function before PCoA analysis. Multivariate analysis of viral community composition under sorting treatment was conducted using PERMANOVA+ [78].

### Heatmap clustering of families and contigs

To construct heatmaps of the abundance of identified families and contigs in each sample, RPKM values were normalized using a log_10_(*x* + 1) transformation and plotted using ClustVis [79]. A Pearson correlation was used to calculate the distance matrix between different viral families.

### Virome analysis in P1 subpopulation

Reads from bulk viral metagenomes were mapped to the annotated viral genomes. MetaSPAdes (v3.15.3) [80], owing to its well virome assembly performance across different assemblers [34, 48], was used to assemble reads from P1. Given the limited random access memory resources available, we subsampled the P1 subvirome to the sequencing depth of 8 million and 4 million forward and reverse reads (equivalent to 36% and 18% of original reads) [81]. After that, the filtered contigs were run in CheckV to assess the quality and completeness of metagenome‐assembled viral genomes [82] and confirmed with VirSorter2 again. DRAMv was then used to identify and annotate viral contigs [81]. Reads from bulk viral metagenomes (PST and SST) and P1 were mapped to the annotated assembled viral genomes.

### Transmission electron microscopy of P1 subpopulations

The sorted particles were concentrated approximately 100 times using the 100‐kDa molecular weight cutoff ultrafiltration centrifugal tubes (Amicon Ultra‐15 Centrifugal Filter Units; Millipore) before viewing under the TEM. To prepare a TEM sample, 20 μl of the concentrated sample was placed on a 200‐mesh carbon‐coated copper grid and the excess liquid was blotted off from the side of the grid with filter paper after 10 min. The grid was negatively stained with 20 μl of gadolinium triacetate (1% wt/wt) for 1 min and the excess stain was blotted off from the side of the grid with filter paper. The grid was left in the dark and dried at room temperature completely before viewing under a JOEL JEM‐2100F TEM.

### Statistical analysis

Statistical tests of the Kruskal–Wallis test and the Mann–Whitney test were performed using GraphPad Prism Version 8.4.0 (GraphPad Software).

## AUTHOR CONTRIBUTIONS

Xiaoqiong Gu, Yi Yang, and Karina Yew‐Hong Gin conceptualized and designed this study; Xiaoqiong Gu prepared virome sample enrichment, nucleic acids extraction, and the next‐generation sequencing. Yi Yang performed flow cytometry sorting. Feijian Mao verified the flow cytometry method using cyanobacteria. Wei Lin Lee and Federica reviewed and edited the draft and graphical abstract. Fang You performed TEM experiment. Xiaoqiong Gu did bioinformatic analysis, with helpful insights from David M. Needham and Charmaine Ng. Hongjie Chen collected and preprocessed wastewater samples. Xiaoqiong Gu, Yi Yang, and Karina Yew‐Hoong Gin wrote the original draft. All authors reviewed and edited the draft. All authors agreed to submit the manuscript, read and approved the final draft, and take full responsibility for its content, including the accuracy of the data.

## CONFLICT OF INTEREST

The authors declare no conflict of interest.

## Supporting information

Supporting information.

## Data Availability

Raw virome data sets were deposited in the NCBI Sequence Read Archive (SRA) under Accession Nos. SRR6853241–SRR6853251. The data and scripts used are saved on GitHub https://github.com/XiaoqiongGu/Gu_2022_Targetedvirome. Supporting Information (figures, tables, scripts, graphical abstract, slides, videos, Chinese translated version, and update materials) may be found in the online DOI or iMeta Science http://www.imeta.science/.
