# Supplementary material for: A comparative study of flow cytometry‐sorted communities and shotgun viral metagenomics in a Singapore municipal wastewater treatment plant
Source: Imeta. 2022 Jul 28;1(3):e39. doi: 10.1002/imt2.39 (PMC10989988; doi:10.1002/imt2.39)
Supplement: Supplementary file 1 — Supporting information. [file IMT2-1-e39-s001.docx]

**Supporting Information**

# A comparative study of flow cytometry sorted communities and shot-gun viral metagenomics in a Singapore municipal wastewater treatment plant

Xiaoqiong Gu^1,2#^, Yi Yang^3#^, Feijian Mao^1^, Wei Lin Lee^2^, Federica Armas^2^, Fang You^1^, David M. Needham^4,5,6^, Charmaine Ng^1^, Hongjie Chen^1,2^, Franciscus Chandra^1^, Karina Yew-Hoong Gin^1,3*^

*^1^Department of Civil and Environmental Engineering, National University of Singapore, Singapore 117576, Singapore*

*^2^ Antimicrobial Resistance Interdisciplinary Research Group, Singapore-MIT Alliance for Research and Technology, Singapore 138602, Singapore*

*^3^ NUS Environmental Research Institute, National University of Singapore, Singapore 138602, Singapore*

*^4^ Monterey Bay Aquarium Research Institute, Moss Landing, CA 95039, USA*

*^5^ GEOMAR Helmholtz Centre for Ocean Research, Ocean EcoSystems Biology Unit, Wischhofstr. 1-3, 24148 Kiel, Germany*

*^6^ Department of Biological Engineering, Massachusetts Institute of Technology, Cambridge, MA 02139, USA*

*^#^These authors contributed equally to this work.*

** Corresponding author: Karina Yew-Hoong Gin*

*Email: ceeginyh@nus.edu.sg*

The number of pages, tables and figures:

Pages: 26

Tables: 8

Figures: 11

SI (A). The primary concentrated samples were mixed with 8% (w/v) of PEG 8000 (Sigma-Aldrich) and 0.3 M NaCl, and adjusted the pH to 7.2. After incubating at 4 °C for 18 hours, the mixture was centrifuged at 14000 g (KUBOTA, Japan) for 45 min to collect the pellet and then resuspended in PBS. To extract viral particles, an equal volume of chloroform (Sigma-Aldrich) was added and centrifuged at 3000 g (KUBOTA) to obtain the supernatant. The supernatant was filtered through a 0.22 μm syringe filter (sartorius, Germany) and further concentrated at 4000 g to a final volume of 0.5-1 mL using an Ultra-15 centrifugal tube (Amicon Merck, Germany) (Gu et al., 2018).

SI (B). Random amplification protocol.

This protocol consists of 2 rounds of enzymatic reactions. In Round A, reverse transcriptase is used for 2 cycles of 1^st^ strand cDNA synthesis with Primer D (GTT TCC CAG TCA CGA TAN NNN NNN NN). Sequenase is used for 2^nd^ strand synthesis. During Round B, the specific Primer E (GTT TCC CAG TCA CGA TA) is used to amplify the templates previously generated.

Round A

1^st^ strand synthesis with RT

1. Add 1ul of 40 pmol/ul Primer D to 5ul extracted viral nucleic acid and 4ul of water to a final volume of 10ul. Heat to 65^o^C for 5 min in a thermocycler and then let cool at room temperature for 5 min.
2. Add 10ul of a master mix consisting of:
   1. 4μl 5X RT buffer (Promega, US)
   2. 1μl 10 mM dNTP (Promega, US)
   3. 1μl RNAseOUT (Invitrogen, US)
   4. 0.5μl DEPC-treated water (Invitrogen, US)
   5. 1.5μl 0.1M DTT (Invitrogen, US)
   6. 2μl SSIII RT (Invitrogen, US)
3. Incubate at 42^o^C for 60 min.

2^nd^ strand synthesis with Sequenase

1. Heat sample to 94^o^C for 2 min (to abort the RT reaction). Rapidly cool to 10^o^C and hold at 10^o^C for 5 min.
2. Add 10ul Sequenase mix for a total reaction volume of 30ul:
   1. 2ul 5X Sequenase buffer (ThermoFisher Scientific)
   2. 7.7ul DEPC-treated water (ThermoFisher, US)
   3. 0.3ul Sequenase (ThermoFisher Scientific)
3. Ramp from 10^o^C to 37^o^C over 8 min.
4. Hold at 37^o^C for 8 min, rapid ramp to 94^o^C and hold for 2 min.
5. Rapid ramp to 10^o^C and hold for 5 min at 10^o^C while adding 1.2ul of diluted Sequenase (1:4 dilution).
6. Ramp from 10^o^C to 37^o^C over 8 min.
7. Hold at 37^o^C for 8 min, ramp to 94^o^C and hold for 8 min (to inactivate Sequenase). Cool to 10^o^C (hold).

Round B: PCR amplification of randomly primed cDNA

1. Prepare a reaction mix
   1. 10ul 10X PCR buffer (Qiagen)
   2. 2ul dNTP mix (Promega, USA)
   3. 1ul Primer E (100 pmol/ul)
   4. 1ul Taq DNA polymerase (Qiagen, Germany)
   5. 80ul DEPC-treated water (Invitrogen, US)
   6. 6ul Round A template
2. Run
   1. 95^o^C, 15 min
   2. 40 cycles of 94^o^C, 30s; 40^o^C, 30s; 50^o^C, 30s; 72^o^C, 1min
3. Run 10ul of PCR product on 1% agarose gel. A visible smear of DNA should be present between 500bp to 1kb. Make sure there is no DNA in the negative control lane.
4. Perform 3 PCR reactions for each sample. Combine and purify using a PCR purification kit (Promega, USA).

Table S1. Merits of fluorescence-activated sorting (FACS) method: application for an effluent from a local wastewater treatment plant and an in-house isolated cyanophage, PA-SR01 (Zhang et al., 2020).

| Sample | Average | Stdv^a^ | RSD^b^ |
| --- | --- | --- | --- |
| Effluent (*n* = 6) | | | |
| P1 | 5.5E+06 | 6.4E+05 | 11.5% |
| P2 | 4.7E+07 | 4.1E+06 | 8.8% |
| P3 | 7.2E+06 | 5.5E+05 | 7.6% |
| P4 | 7.8E+06 | 3.2E+05 | 4.0% |
| P5 | 4.4E+06 | 2.6E+05 | 5.8% |
| Cyanophage (*n* = 8) | | | |
| Phage | 1.7E+07 | 6.0E+05 | 3.6% |

^a^ stdv: standard deviation.

^b^ RSD: relative standard deviation.

Table S2. Statistics of virome datasets obtained from wastewater samples from the primary settling tank (PST) and the secondary settling tank (SST). PST and SST samples were enriched by hollow fiber ultrafiltration followed by polyethylene glycol (PEG) precipitation and Amicon concentration. P1 to P4 and S1 to S5 are FACS-sorted subpopulations of raw wastewater samples from the PST and SST respectively.

| Sample ID | Total raw PE reads | Total trimmed PE reads | Mapped reads | Mapped reads rates |
| --- | --- | --- | --- | --- |
| FACS sub-populations | | | | |
| P1 | 56359024 | 24165244 | 12047848 | 49.9% |
| P2 | 53782516 | 22479352 | 10741680 | 47.8% |
| P3 | 53941180 | 22714660 | 9763316 | 43.0% |
| P4 | 43168304 | 17421786 | 8959596 | 51.4% |
| S1 | 42795128 | 18769334 | 9854442 | 52.5% |
| S2 | 52833216 | 22306822 | 15525964 | 69.6% |
| S3 | 44480564 | 18926678 | 10368368 | 54.8% |
| S4 | 41517364 | 17865162 | 10600478 | 59.3% |
| S5 | 56497576 | 25599698 | 15888206 | 62.1% |
| Hollow fiber ultrafiltration followed by PEG precipitation and Amicon concentration | | | | |
| PST | 51045040 | 21742862 | 9355042 | 43.0% |
| SST | 52889108 | 23115600 | 10484232 | 45.4% |

Table S3. Percentage of contigs annotated to known viral, bacterial, archaea and eukaryota sequences in the current NCBI non-redundant protein database (updated Jan 2018).

| Samples ID | # of mapped reads | # of mapped reads to contigs annotated to known viral sequences | % of mapped reads to contigs annotated to known bacterial sequences | # of mapped reads to contigs annotated to known archaea sequences | % of mapped reads to contigs annotated to known eukaryota sequences | % of mapped reads to contigs annotated as no hits |
| --- | --- | --- | --- | --- | --- | --- |
| FACS sub-populations | | | | | |  |
| P1 | 12047848 | 3.20% | 42.90% | 0.05% | 13.70% | 40.15% |
| P2 | 10741680 | 2.60% | 42.40% | 0.00% | 14.40% | 40.60% |
| P3 | 9763316 | 2.50% | 40.00% | 0.00% | 14.80% | 42.70% |
| P4 | 8959596 | 1.40% | 27.90% | 0.00% | 21.50% | 49.20% |
| S1 | 9854442 | 2.30% | 64.30% | 0.04% | 9.30% | 24.06% |
| S2 | 15525964 | 1.60% | 83.40% | 0.02% | 3.50% | 11.48% |
| S3 | 10368368 | 1.70% | 68.40% | 0.03% | 5.80% | 24.07% |
| S4 | 10600478 | 1.70% | 73.70% | 0.01% | 7.20% | 17.39% |
| S5 | 15888206 | 1.60% | 76.60% | 0.03% | 5.50% | 16.27% |
| Average | 11527766 | 2.10% | 57.70% | 0.02% | 10.60% | 29.58% |
| Bulk viral metagenomics | | | | | |  |
| PST | 9355042 | 56.60% | 27.30% | 0.26% | 3.60% | 12.24% |
| SST | 10484232 | 48.30% | 29.10% | 0.40% | 1.40% | 20.80% |
| Average | 9919637 | 52.50% | 28.20% | 0.33% | 2.50% | 16.47% |

Table S4. Virion size, structure and nucleic acids in identified viral families.^*^

| Taxonomy | Virion size | Genome size | Enveloped | DNA/RNA |
| --- | --- | --- | --- | --- |
| Alloherpesviridae | 150-200 nm | 134-248 kb, linear | Enveloped | dsDNA |
| Alphatetraviridae | 40 nm | 6.5 kb, linear | Non-enveloped | ssRNA |
| Ascoviridae | 130 nm long, 200-400 nm wide | 156-186 kb, circular | Enveloped | dsDNA |
| Flaviviridae | 40-60 nm | 9.7-12 kb, linear | Enveloped | ssRNA |
| Nyamiviridae | 100-130 nm | 11.6 kb, linear | Enveloped | ssRNA |
| Papillomaviridae | 60 nm | 8 kb, circular | Non-enveloped | dsDNA |
| Secoviridae | 25-30 nm | 4-8 kb, linear | Non-enveloped | ssRNA |
| Sphaerolipoviridae | 50-80nm | 30 kb, linear; 16-19 kb circular | Non-enveloped | dsDNA |
| Tectiviridae | 66 nm, spikes 20 nm | 15 kb, linear | Non-enveloped | dsDNA |
| Totiviridae | 40 nm | 4.6-6.7 kb, linear | Non-enveloped | dsRNA |
| Astroviridae | 35 nm | 6.8-8kb, linear | Non-enveloped | ssRNA |
| Parvoviridae | 18-26 nm | 4-6 kb linear | Non-enveloped | ssDNA |
| Picobirnaviridae | 35-40 nm | 4 kb, linear | Non-enveloped | dsRNA |
| Nodaviridae | 30 nm | 4.5 kb, linear | Non-enveloped | ssRNA |
| Iridoviridae | 120-350 nm in diameter polyhedral | 140-303 kb, linear | Enveloped or non-enveloped | dsDNA |
| Leviviridae | 26 nm | 3.4-4.3 kb, linear | Non-enveloped | ssRNA |
| Polydnaviridae | 34–40 nm with variable length (8-150 nm) | 2.0-31 kb, circular | Enveloped | dsDNA |
| Alphaflexiviridae | 470-800 nm*12-13 nm rod | 5.4-9 kb, linear | Non-enveloped | ssRNA |
| Microviridae | 30 nm | 4.4-6.1 kb, circular | Non-enveloped | ssDNA |
| Geminiviridae | 38 nm*22 nm | 2.5-3.0 or 4.8-5.6 kb, circular | Non-enveloped | ssDNA circular |
| Virgaviridae | 20-25 nm*300 nm | 8.2-11.3kb, linear | Non-enveloped | ssRNA |
| Picornaviridae | 30 nm | 7.1-8.9 kb, linear | Non-enveloped | RNA |
| Poxviridae | 220-450 nm long, 140-260 nm wide | 130-375 kb,linear | Enveloped | dsDNA brick-shaped or ovoid |
| Inoviridae | 7 nm in diameter, 700-2000 nm in length | 4.5-8 kb, circular | Non-enveloped | ssDNA rod |
| Myoviridae | 60 nm in diameter, head-tail structure | 33-244 kb, linear | Non-enveloped | dsDNA |
| Nimaviridae | 275*120 nm | 300 kb, circular | Enveloped | dsDNA |
| Mimiviridae | 400 nm | 1200 kb, linear |  | dsDNA |
| Baculoviridae | 21*260 nm | 80-180 kb, circular | Enveloped | dsDNA |
| Marseilleviridae | 250 nm | 372 kb, circular |  | dsDNA |
| Tombusviridae | 28-34 nm | 4-5.4 kb, linear | Non-enveloped | ssRNA |
| Circoviridae | 20 nm | 1.8-3.8 kb, circular | Non-enveloped | ssDNA |
| Phycodnaviridae | 100-220 nm | 100-560 kb, linear | Enveloped | dsDNA |
| Herpesviridae | 150-200 nm | 120-240 kb, linear | Enveloped | dsDNA |
| Retroviridae | 80-100 nm | 7-11 kb | Enveloped | ssRNA |
| Podoviridae | 60 nm in diameter, head-tail structure | 40-42 kb, linear | Non-enveloped | dsDNA |
| Siphoviridae | 60 nm in diameter, head-tail structure | 50 kb, linear |  |  |
| Caliciviridae | 27-40 nm | 7.3-8.3 kb, linear | Non-enveloped | ssRNA |
| Dicistroviridae | 30 nm | 8.5-10.2kb, linear | Non-enveloped | ssRNA |

* Source: ViralZone website: www.expasy.org/viralzone, Swiss Institute of Bioinformatics

Table S5. Highly abundant taxonomy affiliated contigs in sorted subpopulations of PST (P1 – P4).

| ContigID/  Contig length(bp) | Taxonomy affiliation  (Megan) | Blastn (NCBInt)  (E-value, identity, query coverage) | Blastx-NCBInr  (E-value, identity, query coverage) | VirSorter/VirFinder (pvalue) | # of mapped reads/% of mapped reads to known viral sequences |
| --- | --- | --- | --- | --- | --- |
| P1 | | | | | |
|  |  |  |  |  |  |
| k141_468300/10136 | Podoviridae; unclassified Podoviridae；Anabaena phage A-4L | No hit | DNA primase/helicase [Anabaena phage A-4L] (1E-32,26%,29%) | Cat 2/0 | 94669/24.7% |
| k141_452771/5522 | Podoviridae; unclassified Podoviridae；Anabaena phage A-4L | No hit | DNA primase/helicase [Anabaena phage A-4L] (1E-32, 26%,29%) | No hit/0 | 63220/16.5% |
| k141_60772/3230 | Podoviridae; unclassified Podoviridae; Anabaena phage A-4L | No hit | YP_009042806.1, Terminase [Anabaena phage A-4L] (1E-65,33%,41%) | Cat 1/0 | 61959/16.2% |
| P2 | | | | | |
| k141_50750/3427^*^ | Inoviridae; Inovirus; Enterobacteria phage M13; Enterobacteria phage f1; | AY754023.1 Enterobacteria phage M13 vector DY3F63 complete sequence (0, 99%, 100%) | [NP_510893.1](https://www.ncbi.nlm.nih.gov/protein/NP_510893.1?report=genbank&log$=prottop&blast_rank=1&RID=H9D6P3UK015), phage assembly protein [Escherichia virus M13] (0, 100%, 30%) | Cat 1/ 0.0057 | 124635/43.9% |
| k141_5779/1435^*^ | Inoviridae; Inovirus; Enterobacteria phage M13; Enterobacteria phage f1; | AY754023.1 Enterobacteria phage M13 vector DY3F63 complete sequence (0, 99%, 100%) | Replication protein [Escherichia virus M13] (0,99%,62%) | No hit/ 0.0041 | 41764/14.7% |
| P3 | | | | | |
| k141_19715/1362 | Siphoviridae | No hit | YP_009210619.1， putative DNA ligase [Pseudomonas phage PaMx28] (2E-10, 56%,13%) | No hit/ 0.6729 | 101840/41.9% |
| k141_50750/3427 | Inoviridae; Inovirus; Enterobacteria phage M13; Enterobacteria phage f1; | AY754023.1 Enterobacteria phage M13 vector DY3F63 complete sequence (0, 99%, 100%) | [NP_510893.1](https://www.ncbi.nlm.nih.gov/protein/NP_510893.1?report=genbank&log$=prottop&blast_rank=1&RID=H9D6P3UK015), phage assembly protein [Escherichia virus M13] (0, 100%, 30%) | Cat 1/ 0.0057 | 23670/9.7% |
| k141_292066/1340 | Mimiviridae | No hit | AGD92472.1, hypothetical protein [Megavirus lba] (3.4E-08, 45.5%, 18%) | Cat 2/ 3.99E-05 | 14995/6.2% |
| k141_5779/1435 | Inoviridae; Inovirus; Enterobacteria phage M13; Enterobacteria phage f1; | AY754023.1 Enterobacteria phage M13 vector DY3F63 complete sequence (0, 99%, 100%) | Replication protein [Escherichia virus M13] (0,99%,62%) | Cat 2/0.0041 | 14807/6.1% |
| P4 | | | | | |
| k141_50750/3427 | Inoviridae; Inovirus; Enterobacteria phage M13; Enterobacteria phage f1; | AY754023.1 Enterobacteria phage M13 vector DY3F63 complete sequence (0, 99%, 100%) | [NP_510893.1](https://www.ncbi.nlm.nih.gov/protein/NP_510893.1?report=genbank&log$=prottop&blast_rank=1&RID=H9D6P3UK015), phage assembly protein [Escherichia virus M13] (0, 100%, 30%) | Cat 1/ 0.0057 | 24848/19.9% |
| k141_292066/1340 | Mimiviridae | No hit | AGD92472.1, hypothetical protein [Megavirus lba] (3.4E-08, 45.5%, 18%) | Cat 2/ 3.99E-05 | 20656/16.5% |
| k141_5779/ 1435 | Inoviridae;Inovirus; Enterobacteria phage M13; Enterobacteria phage f1; | AY754023.1 Enterobacteria phage M13 vector DY3F63 complete sequence (0, 99%, 100%) | Replication protein [Escherichia virus M13] (0,99%,62%) | No hit/ 0.0010 | 20289/16.2% |

Table S6. Highly abundant taxonomy affiliated contigs in sorted subpopulations of SST (S1 – S5).

| ContigID | Taxonomy affiliation  (Megan) | Blastn (NCBInt)  (E-value, identity, query coverage) | Blastx-NCBInr  (E-value, identity, query coverage) | VirSorter/VirFinder | # of mapped reads / % of mapped reads to known viral sequences |
| --- | --- | --- | --- | --- | --- |
| S1 |  |  |  |  |  |
| k141_50750/3427 | Inoviridae; Inovirus; Enterobacteria phage M13; Enterobacteria phage f1; | AY754023.1 Enterobacteria phage M13 vector DY3F63 complete sequence (0, 99%, 100%) | [NP_510893.1](https://www.ncbi.nlm.nih.gov/protein/NP_510893.1?report=genbank&log$=prottop&blast_rank=1&RID=H9D6P3UK015), phage assembly protein [Escherichia virus M13] (0, 100%, 30%) | Cat 1/ 0.0057 | 18775/8.2% |
| k141_19715/ 1362 | Siphoviridae | No hit | YP_009210619.1， putative DNA ligase [Pseudomonas phage PaMx28] (2E-10, 56%,13%) | No hit/ 0.6729 | 12082/5.2% |
| k141_5779/ 1435 | Inoviridae; Inovirus; Enterobacteria phage M13; Enterobacteria phage f1; | V00604.2 Phage M13 genome (0, 99.56%, 100%)  J02448.1 Enterobacteria phage f1 complete genome (0, 99.12%, 100%) | Replication protein [Escherichia virus M13] (0,99%,62%) | No hit/ 0.6996 | 8587/3.7% |
| S2 | | | | | |
| k141_50750/3427 | Inoviridae; Inovirus; Enterobacteria phage M13; Enterobacteria phage f1; | V00604.2 Phage M13 genome (0, 99.16%, 100%)  J02448.1 Enterobacteria phage f1 complete genome (0, 98.69%, 100%) | [NP_510893.1](https://www.ncbi.nlm.nih.gov/protein/NP_510893.1?report=genbank&log$=prottop&blast_rank=1&RID=H9D6P3UK015), phage assembly protein [Escherichia virus M13] (0, 100%, 30%) | Cat 1/ 0.0057 | 23722/9.7% |
| k141_259161/9429 | Other viruses | No hit | YP_009210619.1, putative DNA ligase [Pseudomonas phage PaMx28] (2E-10, 56%,13%) | Cat 2/ 0.2153 | 14528/6.0% |
| k141_49470/7108 | Mimiviridae | No hit | YP_004894585.1, putative dTDP-d-glucose 4 6-dehydratase [Megavirus chiliensis] (9.4E-43, 34.4%, 14%) | No hit/  0.3208 | 13982/5.7% |
| k141_19715/ 1362 | Siphoviridae | No hit | YP_009210619.1， putative DNA ligase [Pseudomonas phage PaMx28] (2E-10, 56%,13%) | No hit/ 0.6729 | 9611/3.9% |
| S3 | | | | | |
| k141_50750/3427 | Inoviridae; Inovirus; Enterobacteria phage M13; Enterobacteria phage f1; | V00604.2 Phage M13 genome (0, 99%, 100%)  J02448.1 Enterobacteria phage f1 complete genome (0, 99%, 100%) | [NP_510893.1](https://www.ncbi.nlm.nih.gov/protein/NP_510893.1?report=genbank&log$=prottop&blast_rank=1&RID=H9D6P3UK015), phage assembly protein [Escherichia virus M13] (0, 100%, 30%) | Cat 1/ 0.0057 | 50578/28.6% |
| k141_19715/ 1362 | Siphoviridae | No hit | YP_009210619.1， putative DNA ligase [Pseudomonas phage PaMx28] (2E-10, 56%,13%) | No hit/ 0.6729 | 12106/6.9% |
| S4 | | | | | |
| k141_50750/3427 | Inoviridae; Inovirus; Enterobacteria phage M13; Enterobacteria phage f1; | V00604.2 Phage M13 genome (0, 99%, 100%)  J02448.1 Enterobacteria phage f1 complete genome (0, 99%, 100%) | [NP_510893.1](https://www.ncbi.nlm.nih.gov/protein/NP_510893.1?report=genbank&log$=prottop&blast_rank=1&RID=H9D6P3UK015), phage assembly protein [Escherichia virus M13] (0, 100%, 30%) | Cat 1/ 0.0057 | 22399/12.5% |
| k141_49470/7108 | Mimiviridae | No hit | YP_004894585.1, putative dTDP-d-glucose 4 6-dehydratase [Megavirus chiliensis] (9.4E-43, 34.4%, 14%) | No hit/ 0.3208 | 16535/9.2% |
| k141_259161/9429 | Other viruses | No hit | YP_009210619.1， putative DNA ligase [Pseudomonas phage PaMx28] (2E-10, 56%,13%) | Cat 2/ 0.2153 | 8343/4.7% |
| k141_19715/ 1362 | Siphoviridae | No hit | YP_009210619.1， putative DNA ligase [Pseudomonas phage PaMx28] (2E-10, 56%,13%) | No hit/ 0.6729 | 7508/4.2% |
| S5 | | | | | |
| k141_50750/3427 | Inoviridae; Inovirus; Enterobacteria phage M13; Enterobacteria phage f1; | V00604.2 Phage M13 genome (0, 99%, 100%)  J02448.1 Enterobacteria phage f1 complete genome (0, 99%, 100%) | [NP_510893.1](https://www.ncbi.nlm.nih.gov/protein/NP_510893.1?report=genbank&log$=prottop&blast_rank=1&RID=H9D6P3UK015), phage assembly protein [Escherichia virus M13] (0, 100%, 30%) | Cat 1/ 0.0057 | 29929/11.4% |
| k141_49470/7108 | Mimiviridae | No hit | YP_004894585.1, putative dTDP-d-glucose 4 6-dehydratase [Megavirus chiliensis] (9.4E-43, 34.4%, 14%) | No hit/ 0.3208 | 24825/9.5% |

Table S7. Contigs of human-related virus.

| Contig ID | Taxonomy | Blastn (Nucleotide level)  E-value, identity, query coverage | Blastx (Amino acid level)  E-value, identity, query coverage |
| --- | --- | --- | --- |
| k141_67896 | Astroviridae; Human astrovirus | KX022687.1, Astrovirus MLB2 isolate MLB2-LIHT putative RNA dependent RNA polymerase and putative serine protease genes, partial cds; and putative capsid protein gene, complete cds (0, 98%, 98%) | APB03098.1, putative RNA dependent RNA polymerase [Astrovirus MLB2] (0, 97%, 98%) |
| k141_257572 | Astroviridae; Human astrovirus | HM450380.1, Astrovirus MLB1 HK05, complete genome (0, 98%, 100%) | BAN57337.1, capsid protein [Astrovirus MLB1] (0, 99%, 60%) |
| k141_481005 | Astroviridae; Human astrovirus | AB823732.1, Astrovirus MLB1 genomic RNA, complete genome, isolate: BtnMLB1-86 (0, 96%, 99%) | ACN44169.1, putative serine protease [Astrovirus MLB1] (0, 99%, 91%) |
| k141_58437 | Adenoviridae; Human adenovirus | Adeno-associated virus isolate CHC3013_AAV.FL.circular, complete genome (0,98%,100%) | Rep 78 protein [Adeno-associated virus] (0, 99%, 82%) |
| k141_373155 | Adenoviridae; Human adenovirus | Adeno-associated virus isolate hu.T17 capsid protein VP1 (cap) gene, complete cds (0, 100%, 100%) | capsid protein VP1 [Adeno-associated virus] (0, 100%, 99%) |
| k141_312032 | Adenoviridae; Human adenovirus | Adeno-associated virus isolate CHC3511_AAV.FL.circular, complete genome (0, 98%, 98%) | Rep 68 protein [Adeno-associated virus] (0, 100%, 94%) |

Table S8. PERMANOVA analysis of the Bray-Curtis dissimilarities for the taxonomy affiliated contigs between sub-populations and bulk viromes.

| Source | df | SS | MS | Pseudo-F | P-value |
| --- | --- | --- | --- | --- | --- |
| So | 1 | 9857.5 | 9857.5 | 5.3109 | 0.013 |
| Res | 9 | 16705 | 1856.1 |  |  |
| Total | 10 | 26562 |  |  |  |


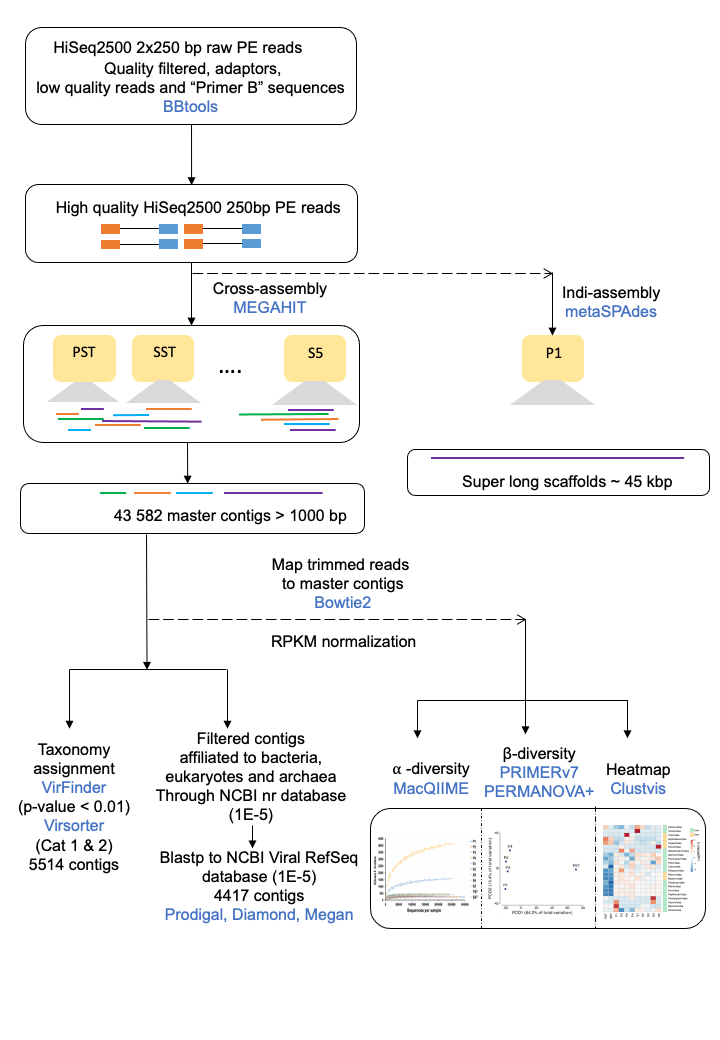
Figure S1. Workflow for the bioinformatics analysis of the sequencing datasets.


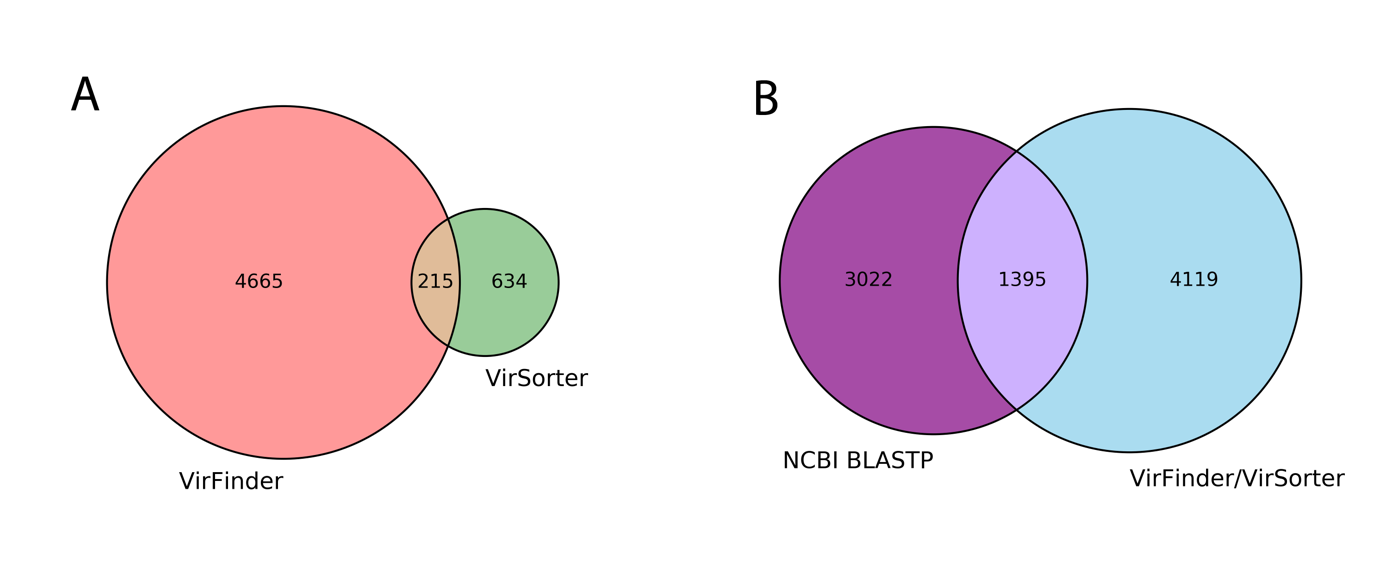


Figure S2. Venn diagram of contigs annotated by (A) VirFinder and VirSorter and (B) NCBI BLASTP and VirFinder/VirSorter


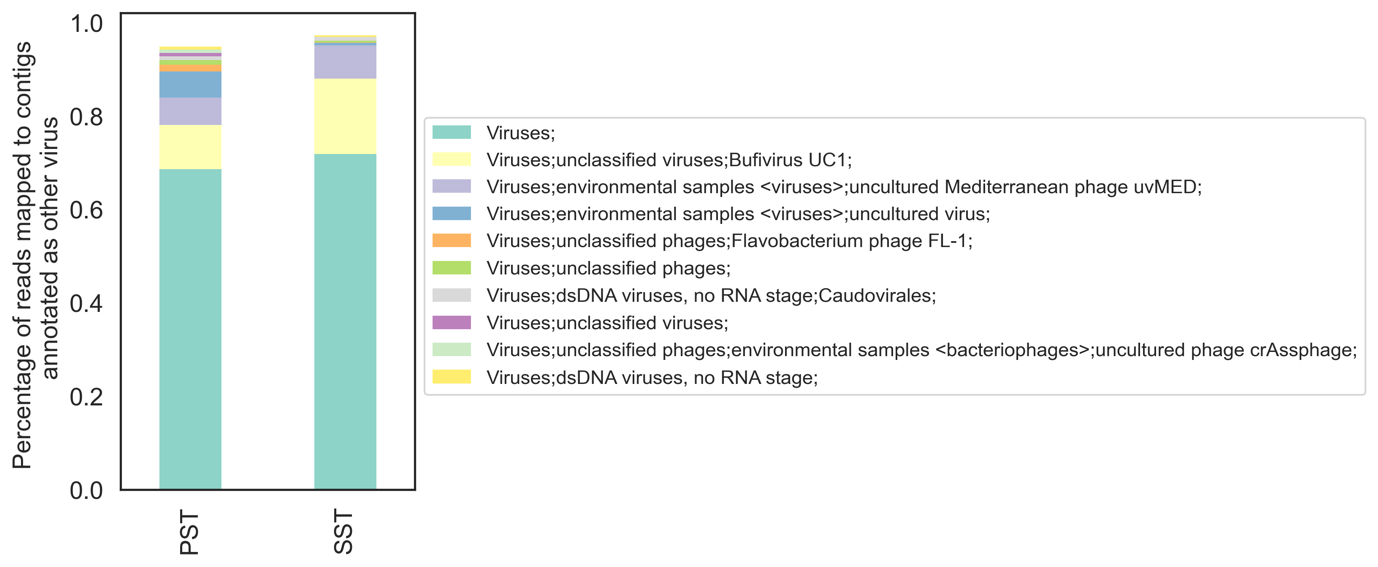


Figure S3. Distribution percentage of mapped reads in PST and SST under the “other viruses” category. Only top 10 hits in PST and SST samples based on the absolute mapped reads were shown.


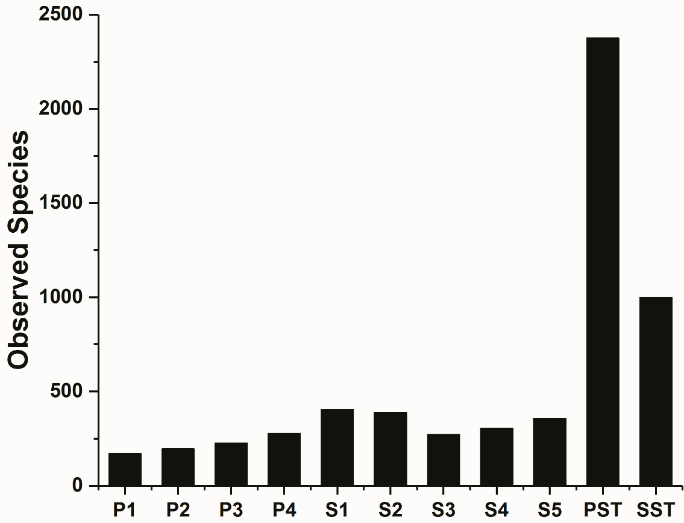

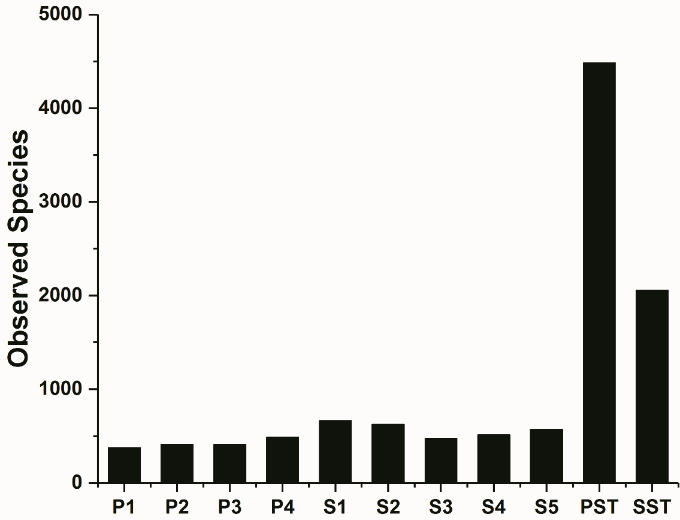


**B**

**Taxonomy affiliated contigs (Megan)**

**VirFinder & VirSorter contigs**

**A**

**B**

**C**

**D**


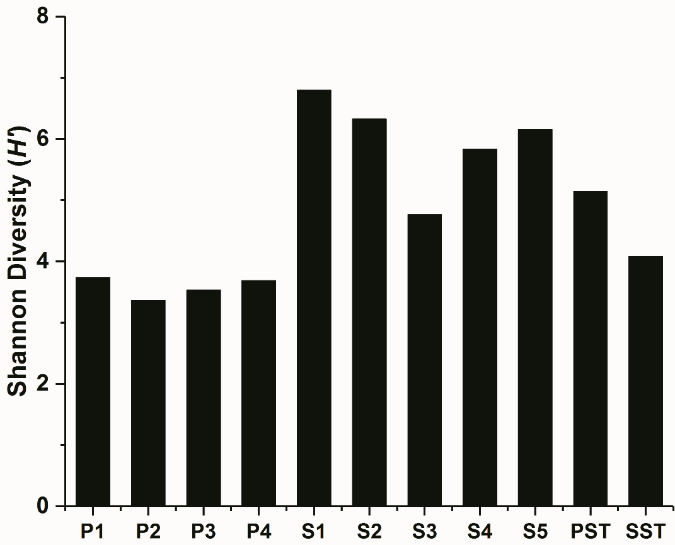

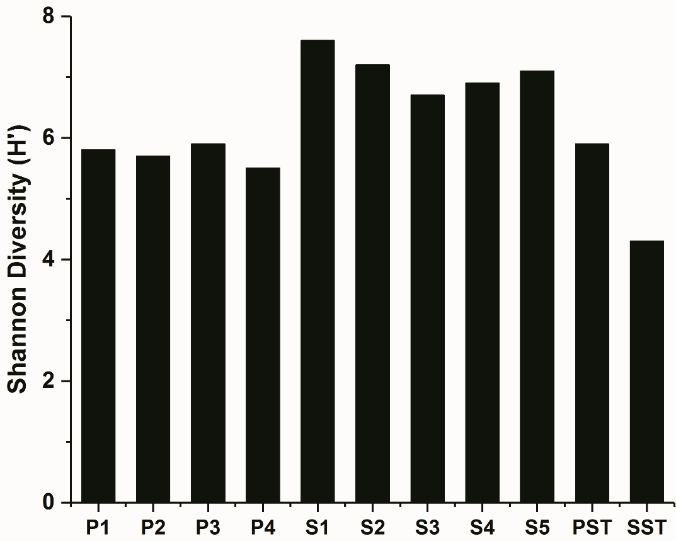
z

Figure S4. Viral contig-based measurements of α-diversity in different samples. Observed Species (A) and Shannon Diversity (B) were quantified based on the contig absolute reads matrix of taxonomy affiliated viruses (matrix was subsampled to 124 811 reads per sample). Observed Species (C) and Shannon Diversity (D) were quantified based on the contig absolute reads matrix of database independent viruses using VirFinder and VirSorter (matrix was subsampled to 426 969 reads per sample). For α-diversity calculated based on VirFinder and VirSorter affiliated contigs (C-D), species richness of 4,482 and 2,056 were observed in PST and SST libraries with Shannon indices of 6.2 and 4.3 respectively. Sorted sub-populations had 375 – 485 species (Shannon index of 5.7) in P1 – P4 and 475 – 664 species (Shannon index of 7.1) in S1 – S5.


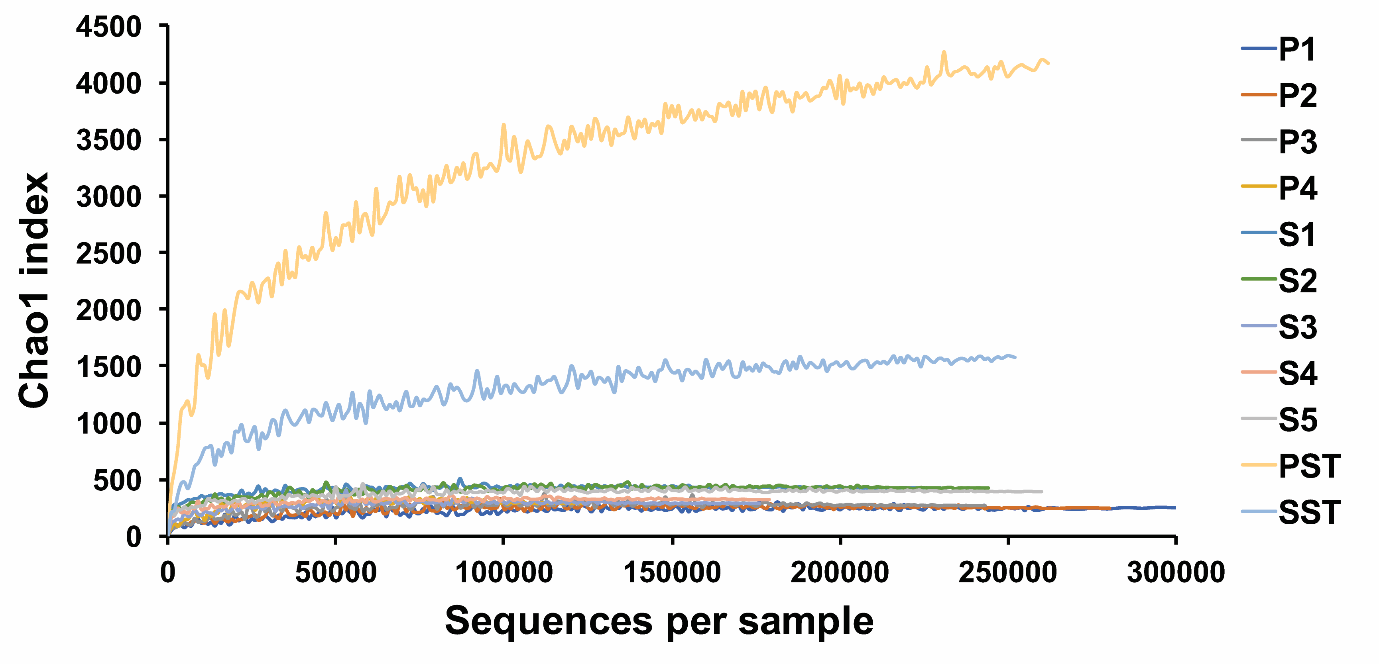


Figure S5. Rarefaction curve for taxonomy affiliated viruses based on Chao1 index. Rarefaction curves of chao1 index appear to plateau with increasing sequencing depth, indicating adequate sequencing depth coverage to investigate dominant viral species.


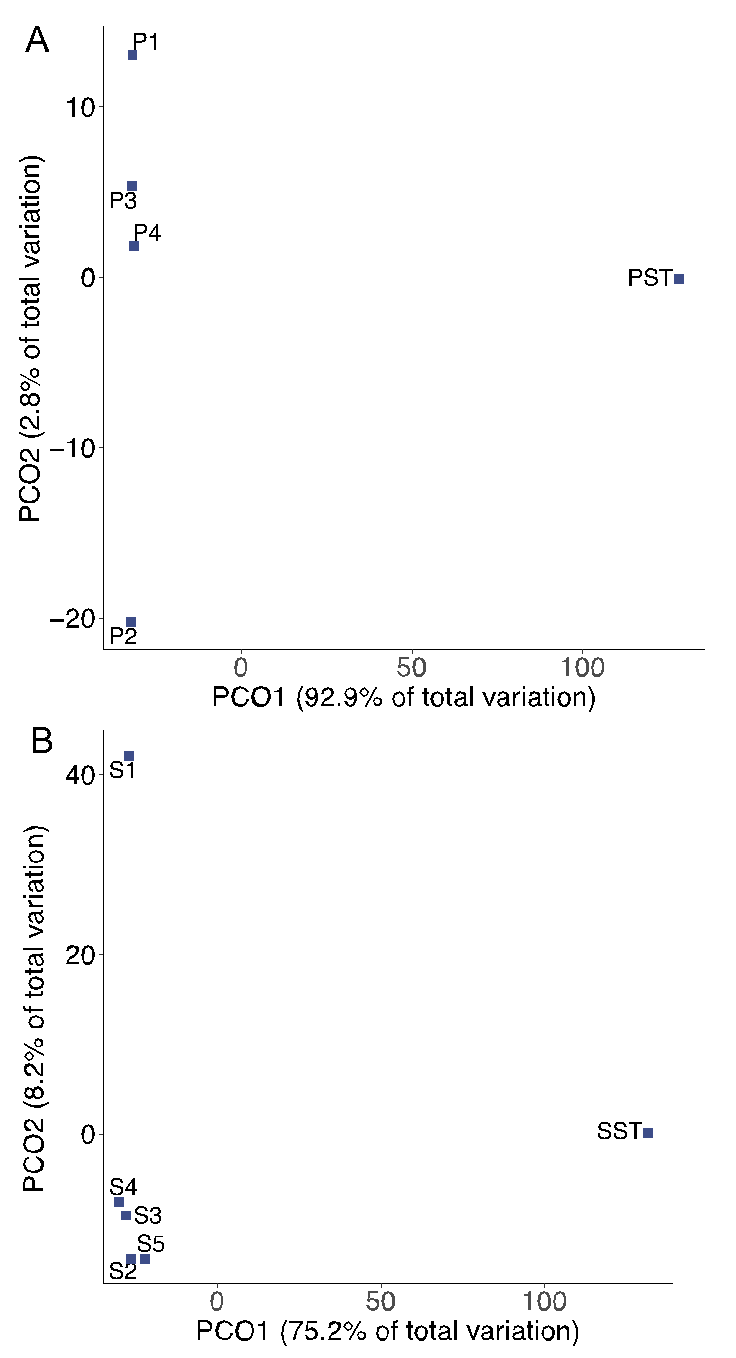


Figure S6. PCoA of VirFinder and VirSorter annotated contigs in (A) PST and sorted sub-populations (P1 – P4) and (B) SST and sorted sub-populations (S1 – S5).


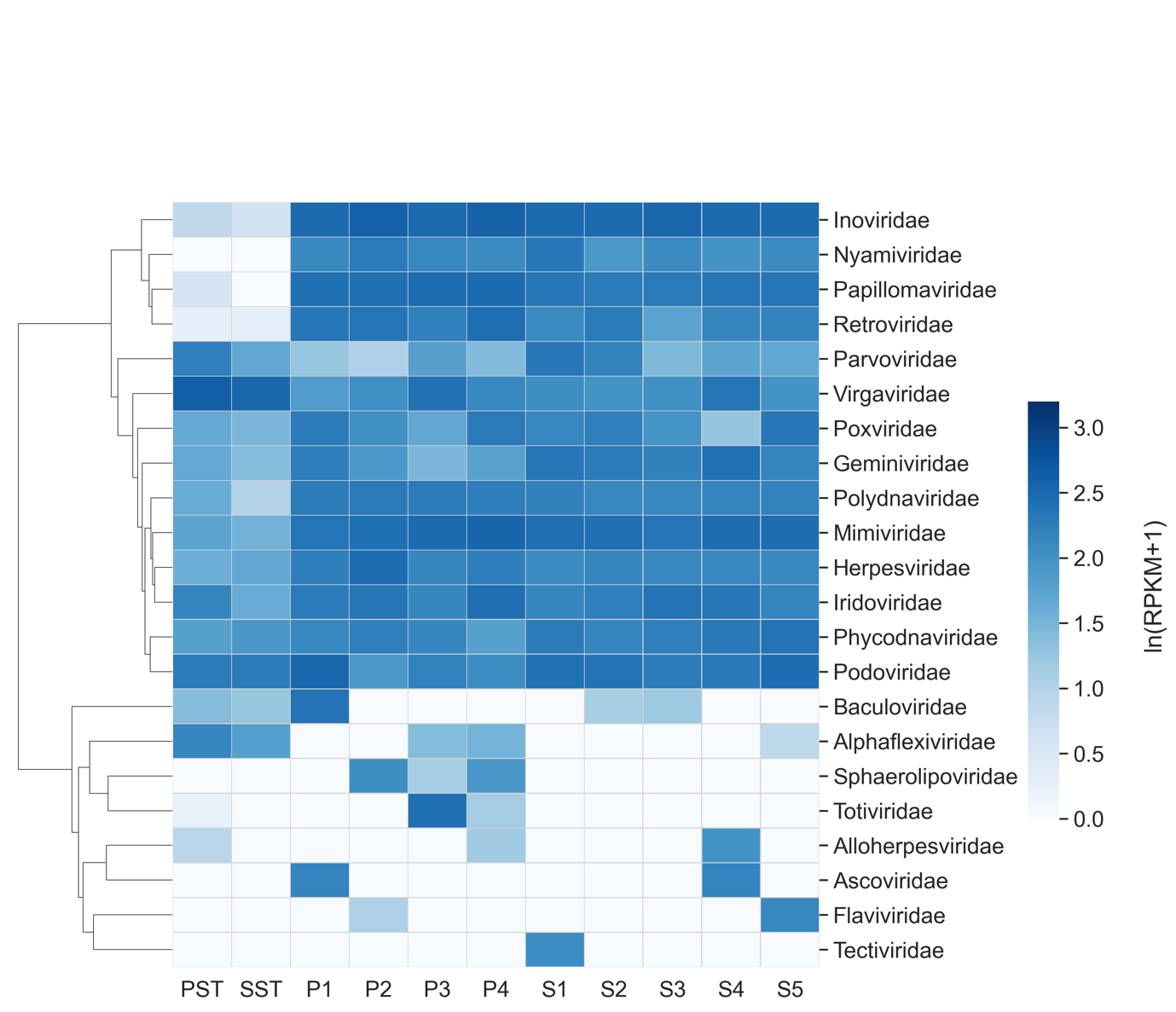


Figure S7. Clustermap of summarized contigs which could not be detected in PST and SST, but were detected in the sorted subpopulations, in original and subpopulation samples. RPKM was transformed using a ln(x+1) function.

Figure S8. Clustermap of summarized contigs which could not be detected in PST and SST, but were detected in the sorted subpopulations, in original and subpopulation samples. RPKM was transformed using a ln(x+1) function. Rows are centered; unit variance scaling is applied to rows. Both rows and columns are clustered using correlation distance and average linkage.

Figure S9. Heatmap of selected high relative abundance viral contigs from each subpopulation (P1 – P4, S1 – S5), in original and subpopulation samples. RPKM was transformed using a ln(x+1) function. The viral contigs were clustered into groups using a correlation analysis. Rows are centered; unit variance scaling is applied to rows. Rows are clustered using correlation distance and average linkage.


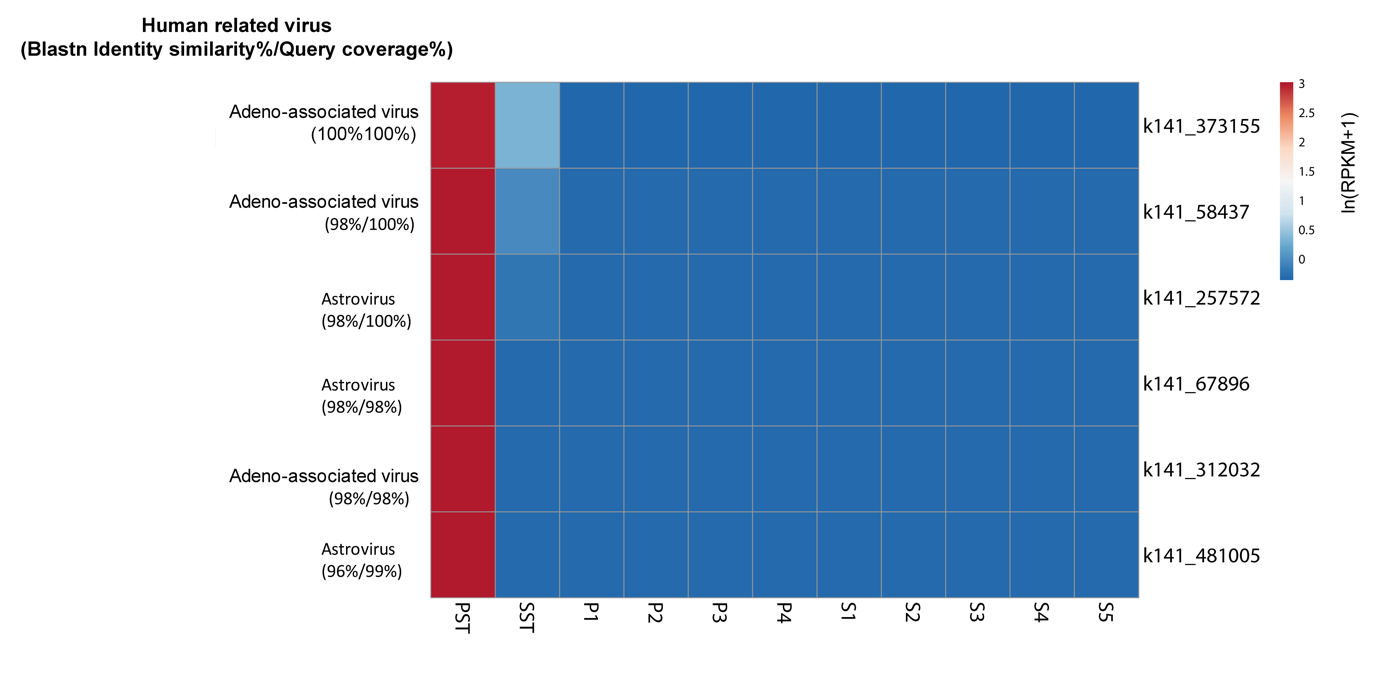


Figure S10. Heatmap of human-related viruses in bulk and subpopulation samples. RPKM was transformed using a ln(x+1) function. The viral contigs were clustered into groups using a correlation analysis. Blastn query coverage and identity similarity are described in the bracket under each annotated viral taxonomy (60% query coverage and 75% identity similarity was used). Rows are centered; unit variance scaling is applied to rows. Rows are clustered using correlation distance and average linkage.


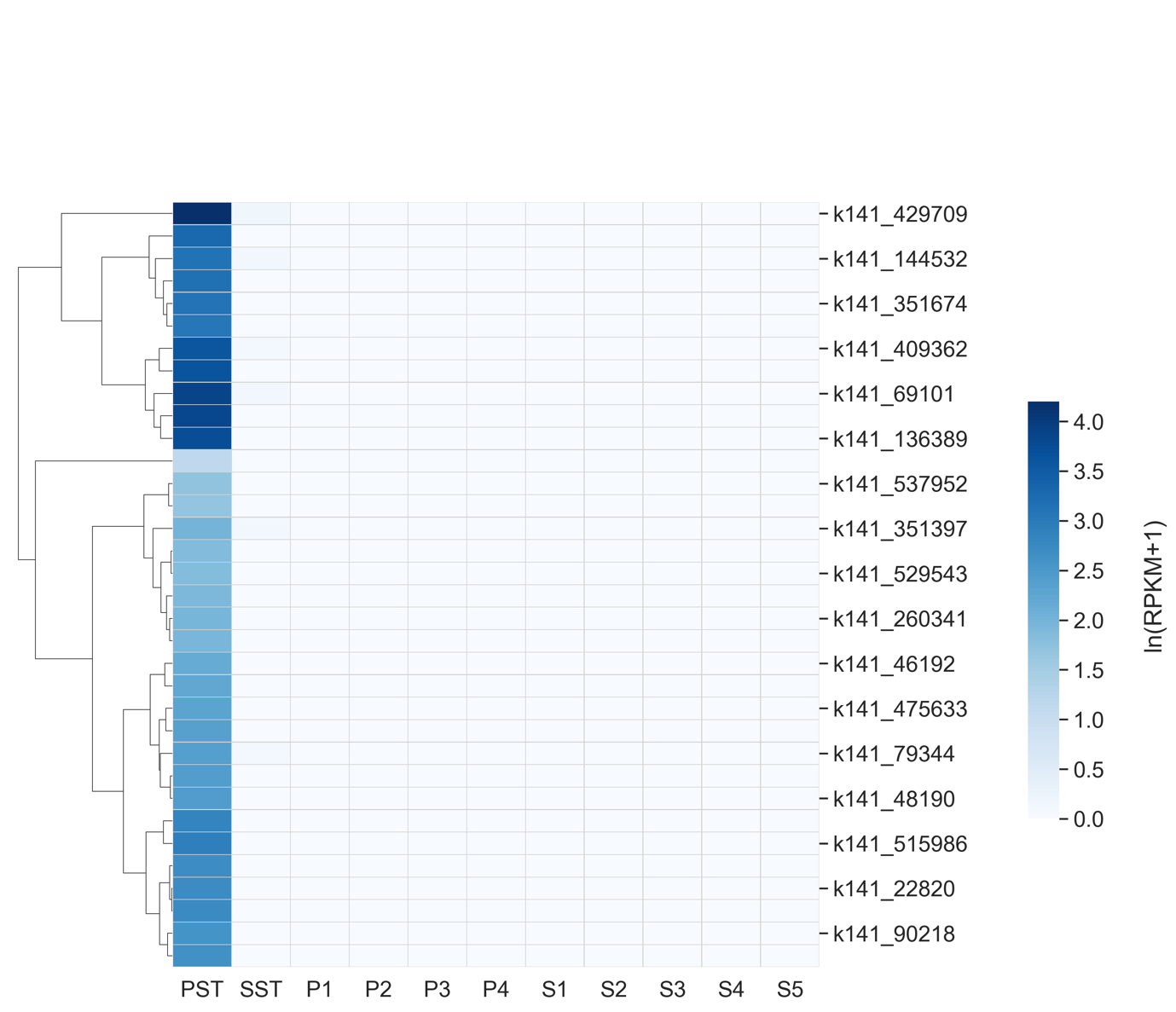


Figure S11. Clustermap of contigs assigned as crAssphages were detected in PST, but nearly undetected in SST and subpopulation samples. RPKM was transformed using a ln(x+1) function.

**Reference**

Gu, X., Tay, Q.X.M., Te, S.H., Saeidi, N., Goh, S.G., Kushmaro, A., Thompson, J.R., Gin, K.Y.-H., 2018. Geospatial distribution of viromes in tropical freshwater ecosystems. Water Res. 137, 220–232. https://doi.org/10.1016/j.watres.2018.03.017

Ng, C., Tan, B., Jiang, X.-T., Gu, X., Chen, H., Schmitz, B.W., Haller, L., Charles, F.R., Zhang, T., Gin, K.Y.-H., 2019. Metagenomic and resistome analysis of a full-scale municipal wastewater treatment plant in Singapore containing membrane bioreactors. Front. Microbiol. 10, 172.

Zhang, D., You, F., He, Y., Te, S.H., Gin, K.Y.-H., 2020. Isolation and Characterization of the First Freshwater Cyanophage Infecting Pseudanabaena. J. Virol. 94. https://doi.org/10.1128/JVI.00682-20
